# The causal association of polyunsaturated fatty acids with allergic disease: A two-sample Mendelian randomization study

**DOI:** 10.3389/fnut.2022.962787

**Published:** 2022-09-09

**Authors:** Yajia Li, Qiangxiang Li, Ziqin Cao, Jianhuang Wu

**Affiliations:** ^1^Department of Dermatology, Xiangya Hospital, Central South University, Changsha, China; ^2^National Clinical Research Center for Geriatric Disorders, Xiangya Hospital, Central South University, Changsha, China; ^3^Ningxia Geriatric Disease Clinical Research Center, People’s Hospital of Ningxia Hui Autonomous Region, Yinchuan, China; ^4^Hunan People’s Hospital, Department of Hunan Institute of Geriatrics, Changsha, China; ^5^Department of Spine Surgery and Orthopaedics, Xiangya Hospital, Central South University, Changsha, China

**Keywords:** polyunsaturated fatty acids, omega-3, omega-6, allergic diseases, Mendelian randomization study

## Abstract

**Objectives:**

Previous studies have reported a potential association of polyunsaturated fatty acids (PUFAs) levels with allergic disease risk and the possible benefit of PUFAs supplementation on allergic disease prevention. This study was performed to estimate the genetic association between PUFAs and allergic diseases using the method of both univariable and multivariable two-sample Mendelian randomization (MR).

**Methods:**

As indicators of the PUFAs levels, we included the omega-3, omega-6, docosahexaenoic acid (DHA), eicosapentaenoic acid (EPA), linoleic acid (LA), and the ratio of omega-6 to omega-3 (omega-6:3). Summarized statistics of genome-wide association studies (GWASs) for these PUFAs were obtained from the United Kingdom Biobank and the Twins United Kingdom cohort. Genetic data relating to allergic diseases, including atopic dermatitis (AD), allergic rhinitis (AR), allergic conjunctivitis (AC), allergic urticaria (AU) and asthma, were accessed from the FinnGen biobank analysis. Odds ratios and 95% CIs were used to express the impact.

**Results:**

The MR results denoted a genetic association between the genetically determined increase in omega-3 levels and the decreased risk of some allergic diseases including AD (OR: 0.863; 95% CI: 0.785 to 0.949; *p* = 3.86E-03), AC (OR:0.720; 95% CI: 0.547 to 0.947; *p* = 1.87E-02) and AU (OR:0.821; 95% CI: 0.684 to 0.985; *p* = 3.42E-02), while omega-6 and DHA level was only found to have negatively correlation with risk of AC with ORs of 0.655 (95% CI: 0.445 to 0.964; *p* = 3.18E-02) and 0.671 (95% CI 0.490 to 0.918; *p* = 1.25E-02), respectively. Omega-6:3 were causally significantly associated with the increased risk of AD (OR:1.171; 95% CI: 1.045 to 1.312; *p* = 6.46E-03) and AC (IVW: OR:1.341; 95% CI: 1.032 to 1.743; *p* = 2.83E-02). After adjustment of age, economic level, BMI, smoking and alcohol behaviors in the multivariable MR analysis, a direct causal protective effect of omega-3 on AD and AC, as well as a direct causal association between DHA and AD were observed. Omega-6:3 was also found to be directly associated with an increased risk of AD and AC. No association was found of EPA or LA with allergic diseases.

**Conclusion:**

Higher PUFA concentrations (omega-3, omega-6, DHA) and lower omega-6:3 ratios were genetically associated with a lower risk of some allergic diseases.

## Introduction

Allergic diseases may involve the respiratory, digestive, skin or other systems and include common conditions such as eczema/atopic dermatitis (AD), allergic asthma, allergic rhino-conjunctivitis (AR/AC)/hay fever/seasonal allergies and allergic urticaria (AU) (1). It is also widely accepted that AD comorbidities extend beyond other allergic conditions, such as AA, AR, AC, and eosinophilic esophagitis, and that allergic diseases follow time-based sequences, suggesting both cutaneous and systemic immune activation (1–3). There has been a noticeable increase in the incidence of allergic disease, which now affects an estimated 20% of the population, making it a public health concern (2, 4, 5). Some allergic conditions with childhood-onset resolve with age, whereas others may persist throughout the lifetime (6), leading to an increased burden on families, society, and healthcare services (7). The rapid escalation of allergic diseases may not be attributed to either genetic or environmental factors (such as lifestyle and diets) alone, and mixed etiology is not fully understood (8). The association between genetic factors and allergic diseases has been extensively studied and some shared susceptibility loci have been identified (9). Large-scale genome-wide association studies (GWAS) and studies of causal roles of genetic susceptibility loci are expected to improve understanding of the prevention and treatment of atopic diseases.

Polyunsaturated fatty acids (PUFAs) of the omega-3 and omega-6 series have been identified by laboratory and epidemiological evidence as having anti-inflammatory and anti-allergy effects (10–13). Especially for omega-3, systematic reviews and meta-analyses have shown the impact of the fish oil-derived omega-3 PUFAs in the primary prevention of allergic disease (14, 15). Indeed, the omega-3 PUFA, docosahexaenoic acid (DHA), and eicosapentaenoic acid (EPA) have been shown to have anti-inflammatory and immunoregulatory properties (13). On the contrast, linoleic acid (LA), one type of omega-6 acid, was found to be linked to increased specific IgE and pro-inflammatory responses among infants (16–18). The ratios of omega-6 to omega-3 PUFAs in some Western diets are found to arise from an equal balance of 1:1 to an unbalanced level of nearly 30:1. The significant changes in PUFAs consumption seem to be paralleled by the increase in the prevalence of atopic and allergic diseases (19), indicating a potential causal relationship between PUFA intake and allergic diseases.

PUFA supplementation has been proposed to prevent allergic disease, and genetic evidence must be considered in establishing the causal effects (20). The current study employed Mendelian randomization (MR) analysis, using instrumental variables (IVs) to explore a causal association of exposure factors with outcomes (21–23). The theory of random distribution of genetic variants within the population, which mimics the randomization process in the assortment of meiosis genetic variants, underpins the approach. An analogy between MR and RCTs may be drawn, with the former less likely to be affected by confounders and reverse causality (24). Two-sample MR analysis relies on genetic effect estimates from two independent summary sets of GWAS to the inference of causal association by comparison with one-sample MR (25). Multivariable MR (MVMR) is an extension of univariable MR and can take the pleiotropy in multiple traits into account. The assumptions of MVMR include the possible effects of genetic variants on multiple measured exposures and the extension of the exclusion restriction and exchangeability assumption (26). Therefore, MVMR can provide a consistent estimator of the direct effect of the primary exposure on the outcome that does not work via the mediator, even when a secondary exposure act as a mediator in the relationship. The current study aimed to infer causal associations between PUFAs (using genetic IVs as proxy) and with risk of atopic disease through a two-sample MR analysis (27).

## Materials and methods

**FIGURE 1:**
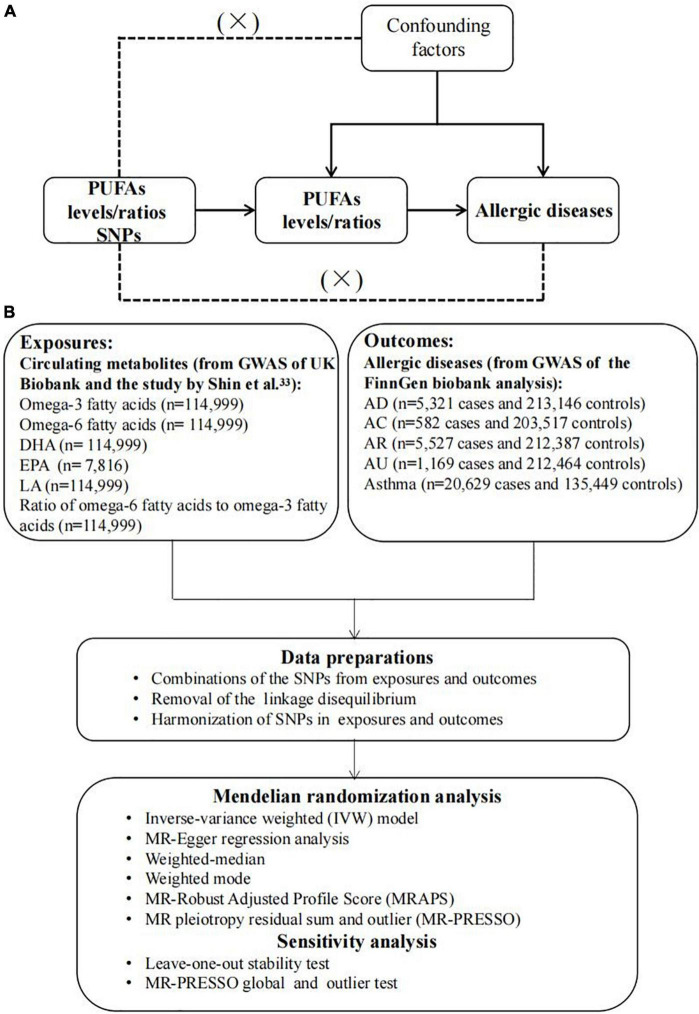
The overview flowchart of hypothesis and schematic design **(A)** Mendelian randomization key hypothesis Diagram. SNPs associated with PUFAs levels/ratios were used as the genetic instruments for investigating the causal effect of PUFA on allergic diseases. Line with arrows indicates that the genetic instruments (SNPs) are associated with the exposure and can only affect the outcome via the exposure. Dashed lines indicate that the genetic instruments (SNPs) are independent of confounders between the results. **(B)** Schematic design for the mendelian randomization analysis.

The overview flowchart of the hypothesis and schematic design is shown in Figure 1. Three principal assumptions were made (Figure 1A) (28): (1) genetic variants were strongly associated with exposure; (2) genetic variants were only associated with the outcome through exposure, and (3) this association was independent of any potential confounders. Publicly available data were used, and no additional informed consent or ethical approval was required. Genetic data were obtained from two large GWAS and, after removing outliers and harmonizing alleles, MR analysis with six different methods and sensitivity analysis was applied to identify causal associations between PUFAs and allergic diseases.

### Data source and selection of genetic instrumental variables

Single-nucleotide polymorphisms (SNPs) were identified and used as IVs from eligible datasets in GWAS Catalog, IEU openGWAS and NealELab. Only GWAS conducted on individuals of European ancestry were included to limit the bias resulting from ethnic confounders. Six main dietary PUFAs indexes were considered in the present study: SNPs for circulating omega-3, omega-6, DHA, EPA, and LA levels, as well as the ratio of omega-6 to omega-3 fatty acids (omega-6:3), were also obtained as instrumental variables of exposure. Genetic risk variants of exposure including omega-3, omega-6, DHA, LA, and omega-6:3 were identified from the Metabolic biomarkers in the United Kingdom Biobank (Nightingale Health 2020). Circulating omega-3 and omega-6 fatty acids, as well as DHA and LA concentrations, were measured from randomly selected EDTA plasma samples by using a targeted high-throughput nuclear magnetic resonance (NMR) metabolomics platform (Nightingale Health Ltd; biomarker quantification version 2020) (29). In total, 121,577 samples were retained for analyses after removing duplicates and observations not passing quality control in the non-fasting plasma samples collected at baseline, and 114,999 samples were retained in the final. Details for measurement technology and applications for the epidemiology of this platform have been previously reviewed (30–32). For the EPA level, it was obtained from the Twins United Kingdom cohort (33), which is an adult twin British registry composed of mostly women recruited from the general United Kingdom population through national media, and the EPA level was measurable in blood using the Metabolon platform. The detailed information was described in the previous studies (34–36). Genetic data relating to AD, AC, AR, AU, and asthma were accessed from the FinnGen biobank analysis (round 5), and diagnoses were based on ICD-10 (Figure 1B).

Summarized statistics of PUFA-related SNPs with genome-wide significance (*p* < 5 × 10^–8^) were designated as alternate IVs. Linkage disequilibrium (LD) was tested within the condition of the clumping algorithm with *r*^2^ = 0.001 and kb = 10,000 to reduce the effect of strong LD. *F* statistics were used to assess the risk of weak instrumental bias with at least 10 being a sufficient level for MR analysis (36, 37). Based on the merged dataset of exposure-outcome, harmonization of effect alleles and subsequent analyses were conducted. Detailed information regarding IVs is presented in Supplementary Tables 1–6.

### Two-sample Mendelian randomization

Primary MR analysis was performed using the inverse-variance weighted (IVW) model, combining Wald estimates of causality for each IV with the assumption of invalid genetic instruments (e.g., a balanced pleiotropy) (38, 39). MR-Egger regression analysis and weighted-median estimator were used to examine any violation of MR assumptions caused by directional pleiotropy (40, 41). The MR-Egger intercept estimates the effect of pleiotropy across genetic variants and provides a relatively robust estimate with the independence of IV validity and an adjusted result via the regression slope (38, 40). A consistent valid estimate could be inferred by a weighted-median estimator if over 50% of instrumental variables were valid (40, 41). The weighted mode-based method infers robust overall causal estimates on the condition that individual estimates were mostly obtained from valid IVs (42). MR-Robust Adjusted Profile Score (MRAPS) was used to derive a more accurate assessment of causal association with ideal independence of IVs (43). In addition, MR pleiotropy residual sum and outlier (MR-PRESSO) was used to detect and correct horizontal pleiotropy by the removal of outliers with *p* < 0.05 and to give a corrected causal effect (44). Cochran’s Q-statistic was used to assess heterogeneity, and a random-effect model was used for subsequent analyses with *p* < 0.05 as a level of significant heterogeneity (45). In MR-PRESSO analysis, heterogeneity and pleiotropy in causal effect estimates were reduced by removing outliers and reassessing causal estimates. If heterogeneity was still significant after removing outliers, all SNPs with a *p*-value < 1 in the MR-PRESSO outlier test were removed. The MR analysis was re-performed with results from the random-effect IVW model being adopted. The number of distributions in the MR-PRESSO analysis was set to 1,000. Additional sensitivity analyses were performed by the exclusion of IVs one at a time (46). Other statistical tools were used to complement IVW and produced wider confidence intervals (CIs) (47). Therefore, IVW results were prioritized, and the MR-Egger was adopted for significant pleiotropy and the MR-PRESSO to detect final outliers. The flow chart of analytical methods used in this MR analysis is shown in Supplementary Figure 1.

In additional analyses, to investigate the direct effects of PUFAs on allergic diseases, MVMR analysis was also performed as an extension of univariable MR allowing the joint detection of causal effects of multiple risk factors (26, 48). Genetic associations between SNPs and age, average total household income, body mass index (BMI), smoking, and alcohol were obtained from a recent GWAS using a United Kingdom Biobank sample of 2,336,260 to 1,3586,591 individuals of European descent. MVMR takes into account the relationships among PUFAs, age, income, BMI, smoking, and alcohol drinking, and the fact that the SNPs selected in the MR analyses are often associated with several phenotypes. Therefore, MVMR was used to evaluate the direct effects of PUFAs independent of the effects of age, income, BMI, smoking, and alcohol assumptions on allergic diseases. The clumping window of r^2^ = 0.001 and kb = 10,000 was also used to reduce the effect of strong LD in all mediators. The combination of all GWAS-significant SNPs with a *P*-value less than 5 × 10^–8^ were extracted from each exposure and were clump for avoiding LD under a window of r^2^ = 0.001 and kb = 10,000. Selected IVs were further analyzed in the multi-variable IVW and MR-Egger models, and a *P*-value < 0.05 was considered independently significant in the MVMR analysis. It should be noted that both IVW and MR-Egger methods could reveal heterogeneity in the analysis, and the results of MR-Egger would be applied when there was pleiotropy detected (26, 49).

### Statistical analysis

Odds ratios and 95% CIs were used to express the impact on allergic disease risk caused by a corresponding unit change in absolute levels of the circulating omega-3, omega-6 and DHA and the ratio omega-6:3. According to the rules of Bonferroni correction for reduction of false positives by multiple tests, a two-sided *p*-value < 0.0083 was considered statistically significant but *p*-values ≥ 0.0083 and < 0.05 were only suggestive of statistical significance. MR analyses were performed using the “TwoSampleMR.” package (version 0.5.6) and Mendelian Randomization (50) (version 0.5.0) packages in R software (version 4.1.2), R Foundation for Statistical Computing, Vienna, Austria). All study results are reported according to STROBE-MR (Strengthening the Reporting of Observational Studies in Epidemiology—Mendelian Randomization) guidelines (51).

## Results

Data regarding SNPs relating to omega-3, omega-6, DHA, LA, EPA, and omega-6:3 exposure are given in Supplementary Tables 1–6. F-statistics for all selected IVs are almost >10, indicating no weak IVs. Details of sensitivity analysis and outliers are shown in Table 1.

**TABLE 1:** Sensitivity analyses of the raw MR analysis and the adjusted MR analysis (adjusted by excluding all outliers and heterogeneous SNPs identified by the MR-PRESSO test).

Exposure	Outcome	nIVs	Heterogeneity test		MR-Egger pleiotropy test		MR-PRESSO global test		MR-PRESSO distorted outlier test		F statistics
			Q (*P*-value)	adjusted Q (*P*-value)	Intercept (*P*-value)	adjusted Intercept (*P*-value)	RSSobs (*P*-value)	adjusted RSSobs (*P*-value)	Outlying SNPs	Heterogeneous SNPs	
Omega-3	AD	46	79.03 (0.0024)	56.32 (0.1201)	0.0026 (0.7093)	0.0067 (0.2817)	83.5102 (0.010)	54.0308 (0.268)	rs11242109	rs144018203, rs3129962	281.89
	AC	48	54.22 (0.2184)	NA	0.0135 (0.4209)	NA	55.6056 (0.280)	NA	None	None	272.84
	Asthma	42	127.85 (0.0000)	41.48 (0.4499)	−0.0135 (0.0055)	−0.0077 (0.0745)	146.0187 (< 0.001)	43.4303 (0.478)	rs11242109, rs174564	rs10184054, rs2394976, rs4860987, rs77960347	135.98
	AR	48	57.22 (0.1459)	NA	−0.0118 (0.0367)	NA	62.7635 (0.156)	NA	None	None	272.84
	AU	48	48.10 (0.4279)	NA	−0.0216 (0.0528)	NA	51.5931 (0.446)	NA	None	None	272.84
Omega-6	AD	50	53.22 (0.3152)	NA	0.0128 (0.1055)	NA	55.0880 (0.309)	NA	None	None	130.06
	AC	50	52.05 (0.3560)	NA	0.0433 (0.0521)	NA	54.6354 (0.334)	NA	None	None	130.06
	Asthma	50	55.46 (0.2444)	NA	−0.0015 (0.7368)	NA	58.3651 (0.216)	NA	None	None	130.06
	AR	50	57.63 (0.1863)	NA	−0.0001 (0.9904)	NA	59.4571 (0.196)	NA	None	None	130.06
	AU	50	39.13 (0.8424)	NA	−0.0122 (0.4298)	NA	41.1063 (0.818)	NA	None	None	130.06
RO63	AD	35	52.55(0.0220)	NA	−0.0038(0.6241)	NA	55.5381 (0.060)	NA	None	None	105.22
	AC	35	33.3804 (0.4978)	NA	0.0140 (0.4342)	NA	34.67916 (0.594)	NA	None	None	105.22
	Asthma	29	119.7397 (0.0000)	30.8934 (0.3218)	0.0135 (0.0361)	−0.0012 (0.8455)	138.0924 (0.001)	32.6248 (0.347)	rs11242109, rs11632618, rs174564	rs2394976, rs4860987, rs7222755	92.45
	AR	35	45.8239 (0.0848)	NA	0.0071 (0.3079)	NA	48.4527 (0.161)	NA	None	None	105.22
	AU	35	40.0812 (0.2185)	NA	0.0288 (0.0311)	NA	44.3980 (0.287)	NA	None	None	105.22
DHA	AD	39	65.37 (0.0091)	40.84 (0.3468)	0.0095 (0.2291)	0.0006 (0.9525)	74.2713 (0.032)	42.9112 (0.333)	rs174564	rs182611493, rs525028	95.32
	AC	42	42.95 (0.3876)	NA	–0.0138 (0.4531)	NA	44.5644 (0.451)	NA	None	None	214.17
	Asthma	37	70.08 (0.0031)	35.10 (0.5113)	−0.0125 (0.0045)	−0.0043 (0.4359)	90.4094 (0.019)	36.4349 (0.557)	rs174564	rs2394976, rs273912, rs4860987, rs77960347	97.74
	AR	42	49.16 (0.1789)	NA	−0.0099 (0.1289)	NA	53.3817 (0.211)	NA	None	None	214.17
	AU	42	46.89 (0.2436)	NA	−0.0113 (0.4028)	NA	47.6418 (0.312)	NA	None	None	214.17
LA	AD	40	58.9374 (0.0431)	33.2374 (0.7295)	0.0138 (0.1832)	0.0168 (0.0626)	62.5178 (0.0410)	35.0954 (0.7310)	rs141469619	rs174564, rs4947302	136.62
	AC	43	48.5985 (0.2244)	NA	0.0402 (0.1340)	NA	51.8960 (0.1980)	NA	None	None	138.80
	Asthma	40	63.7698 (0.0167)	38.0106 (0.5149)	0.0017 (0.7751)	–0.0026 (0.6079)	68.9051 (0.0140)	40.7977 (0.4960)	rs693	rs77960347, rs174564	138.80
	AR	43	49.1533 (0.2084)	NA	−0.0011 (0.9023)	NA	51.2625 (0.2090)	NA	None	None	138.80
	AU	43	43.8677 (0.3923)	NA	0.0000 (0.9982)	NA	46.4538 (0.3920)	NA	None	None	138.80
EPA	AD	7	11.1523 (0.0838)	NA	0.0866 (0.0319)	NA	19.3726 (0.1340)	NA	None	None	9.91
	AC	7	3.7916 (0.7048)	NA	0.0525 (0.5606)	NA	4.6240 (0.7750)	NA	None	None	9.91
	Asthma	7	9.8296 (0.1320)	NA	−0.0211 (0.3652)	NA	21.5129 (0.1440)	NA	None	None	9.91
	AR	7	5.1788 (0.5211)	NA	0.0149 (0.6195)	NA	6.4895 (0.6070)	NA	None	None	9.65
	AU	7	4.1837 (0.6518)	NA	0.0592 (0.3644)	NA	5.2409 (0.7160)	NA	None	None	9.91

As the Supplementary Figure 1 showed, for the process of adjustment, we firstly did a raw MR analysis and got an uncorrected causal evaluation. Then, MR-PRESSO global and Outliers test was performed to find unstable SNPs, and an adjusted MR analysis was performed again after removing all unstable SNPs, and the heterogeneity, pleiotropy and causal effect values were re-evaluated. MR: Mendelian randomization analysis; nIVs: Number of instrumental variables; NA: Not applicable; AD: atopic dermatitis; AC: Atopic conjunctivitis; AR: Allergic rhinitis; AU: Allergic urticaria; Omega-3: Omega-3 fatty acids; Omega-6: Omega-6 fatty acids; DHA: Docosahexaenoic acid.

### Causal effects of omega-3/omega-6 on allergic diseases

The association of omega-3 with AD risk showed no evidence of directional pleiotropy but significant heterogeneity, according to Cochran’s Q test (Q = 79.029; *p* = 0.002), but the removal of 3 outliers abolished heterogeneity. The genetically determined per unit increase in circulating omega-3 was associated with decreased risk of AD (outlier-corrected: OR: 0.863; 95% CI: 0.785 to 0.949; *p* = 3.86E-03).

**FIGURE 2:**
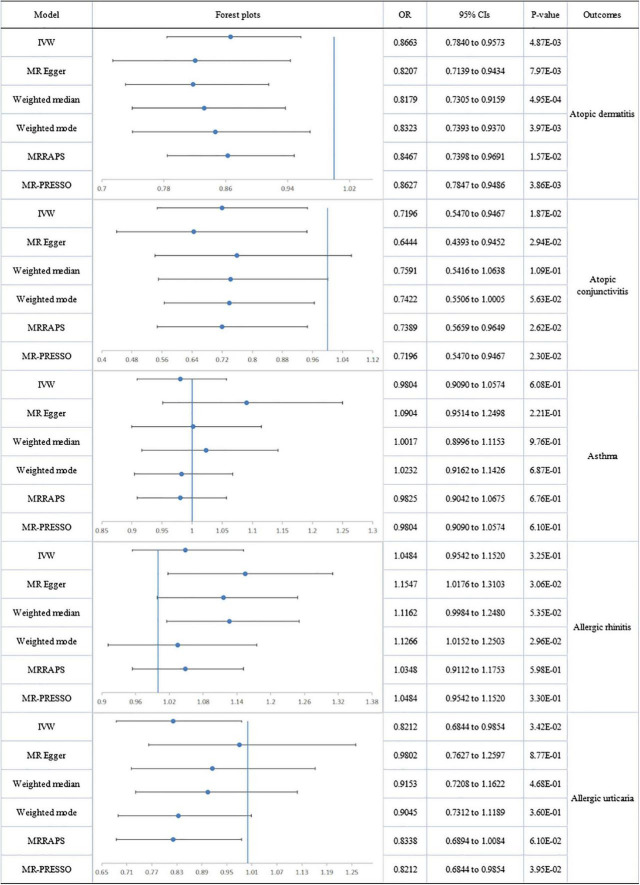
The forest plot of univariable Mendelian randomization analyses exploring associations between omega-3 fatty acids and risk of allergic diseases using different Mendelian randomization statistical models OR: odds ratio; CIs: confidence intervals.

No directional pleiotropy or heterogeneity was found for the association of circulating omega-3 on AC, AR or AU. A genetically determined increase in plasma omega-3 levels produced a trend with suggestive significance for decreased risk of AC (IVW-fixed: OR:0.720; 95% CI: 0.547 to 0.947; *p* = 1.87E-02) and AU (IVW-fixed: OR:0.821; 95% CI: 0.684 to 0.985; *p* = 3.42E-02), but no association was found between omega-3 and AR. Pleiotropy, assessed by MR-Egger regression (intercept = −0.014; *p* = 0.005), and heterogeneity (Q = 127.849; *p* = 2.08E-09) were analyzed for the relationship between circulating omega-3 and asthma, but after removal of six outliers, there was still no significant association (Figure 2).

**FIGURE 3:**
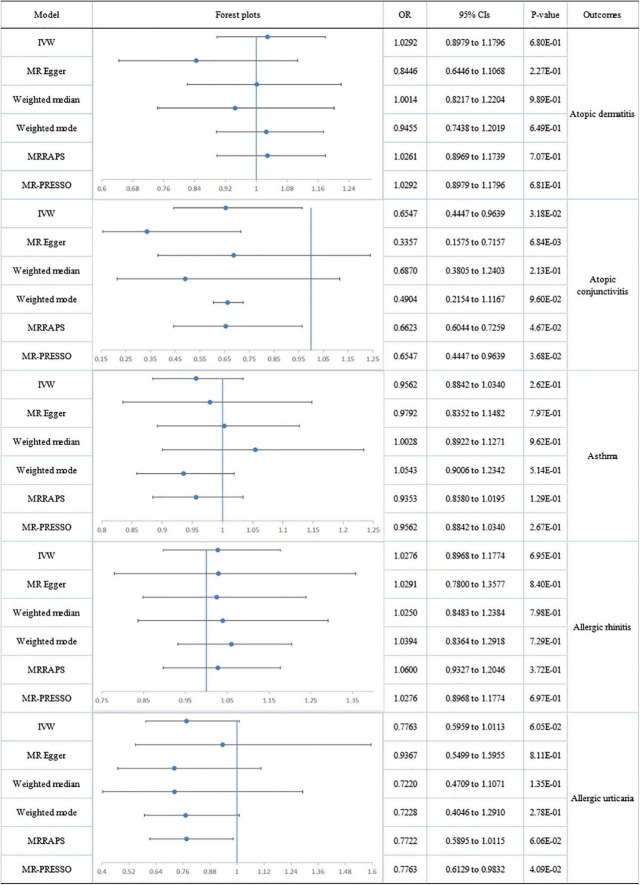
The forest plot of univariable Mendelian randomization analyses exploring associations between omega-6 fatty acids and risk of allergic diseases using different Mendelian randomization statistical models OR: odds ratio; CIs: confidence intervals.

No directional pleiotropy or significant heterogeneity was found for the analysis of circulating omega-6 levels and atopic diseases. A suggestively significant association emerged between omega-6 level and AC (IVW-fixed: OR:0.655; 95% CI: 0.445 to 0.964; p = 3.18E-02), but no relationship with other allergic diseases was found (Figure 3).

### Causal effects of docosahexaenoic acid, eicosapentaenoic acid, and linoleic acid on allergic diseases

**FIGURE 4:**
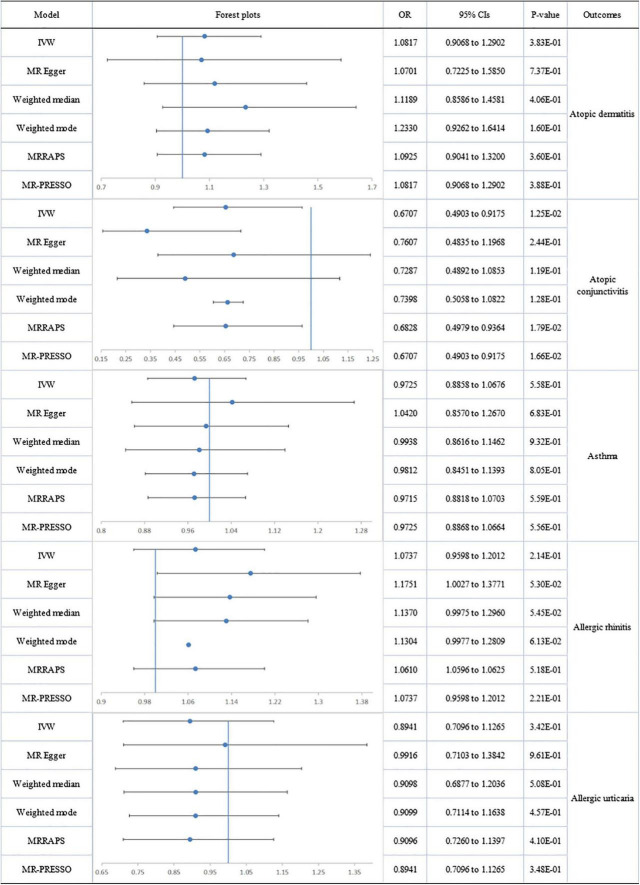
The forest plot of univariable Mendelian randomization analyses exploring associations between docosahexaenoic acid and allergic diseases risk using different Mendelian randomization statistical models OR: odds ratio; CIs: confidence intervals.

The association between DHA and AD showed heterogeneity, detected by Cochran’s Q test (Q = 65.374; *p* = 0.009), but no directional pleiotropy. The removal of 4 outliers abolished heterogeneity, allowing the adoption of a fixed-effect model. No genetic association was found between circulating DHA and AD. No heterogeneity or directional pleiotropy emerged from the analyses of DHA association with AC, AR or AU. A suggestively significant association was only revealed between the DHA level and decreased risk of AC (IVW-fixed: OR:0.671; 95% CI 0.490 to 0.918; *p* = 1.25E-02). Pleiotropy, by MR-Egger regression (intercept = −0.013; *p* = 0.004), and heterogeneity (Q = 70.079; *p* = 0.003) were assessed in the analysis of DHA and asthma but after removal of four outliers (rs2394976, rs273912, rs4860987, rs77960347), no significant association was found (Figure 4). There was no significant association of LA and EPA with allergic diseases (Supplementary Figures 2, 3).

### Causal effects of the ratio of omega-6 to omega-3 on allergic diseases

**FIGURE 5:**
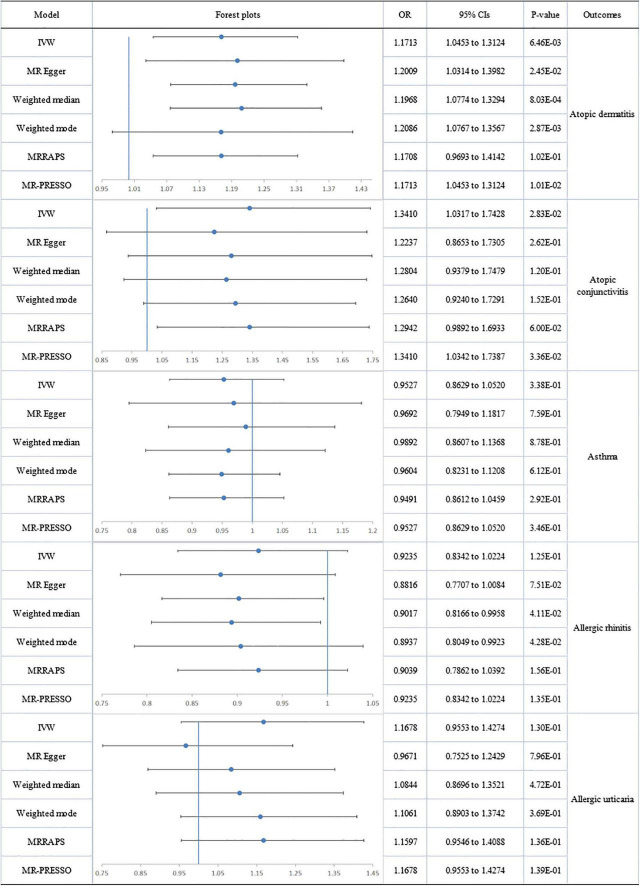
The forest plot of univariable Mendelian randomization analyses exploring associations between ratio of omega-6 fatty acids and omega-3 fatty acids to allergic diseases risk using different Mendelian randomization statistical models OR: odds ratio; CIs: confidence intervals.

No heterogeneity or directional pleiotropy was found for the analyses of omega-6:3 on allergic diseases except for asthma and AU. Using the fixed-effect IVW model, circulating omega-6:3 was found to be significantly associated with an increased risk of AD (IVW-fixed OR:1.171; 95% CI: 1.045 to 1.312; *p* = 6.46E-03) and a suggestively significant association with increased risk of AC (IVW: OR:1.341; 95% CI: 1.032 to 1.743; *p* = 2.83E-02) was also found. There was no impact on AR. Significant heterogeneity and pleiotropy were detected respectively by Cochran’s Q test (Q = 119.740; *p* = 1.76E-11) and MR-Egger regression (intercept = 0.014; *p* = 0.036) for analysis of omega-6:3 and asthma. However, after the removal of six outliers, no significant association remained between omega-6:3 and asthma. Significant pleiotropy was detected by MR-Egger regression (intercept = 0.029; *p* = 0.031) for analysis of omega-6:3 and AU but the MR-PRESSO global test reported no evident pleiotropy (RSSobs = 44.398; *p* = 0.287). Therefore, the negative association between omega-6:3 with AU (MR-Egger: OR: 0.967; 95% CI: 0.753 to 1.243; *p* = 7.96E-01) should be interpreted with caution (Figure 5).

A forest plot of the causal estimates of PUFAs on allergic diseases is presented in Figures 2–5. Overall, the consistency of effect sizes across different methods indicates that confidence may be put in the results of each analysis. The corresponding scatter plots for the MR analysis are shown in Supplementary Figures 4–9.

The leave-one-out stability tests conducted by excluding a single SNP at a time are detailed in the Supplementary Figures 10–15. Risk estimates of genetically predicted omega-6 levels and omega-6:3 ratios for allergic diseases did not change substantially after excluding one SNP at a time, indicating that it was unlikely that potential driving SNPs were causing bias to the causal association. However, the removal of rs174564 from the two analyses of omega-3 and DHA levels on risk of AR, caused a distinct change in risk estimates, indicating that this instrumental variable severely affected the outcome variable. Therefore, these particular results should be interpreted with caution.

### Multivariable MR analyses

**FIGURE 6:**
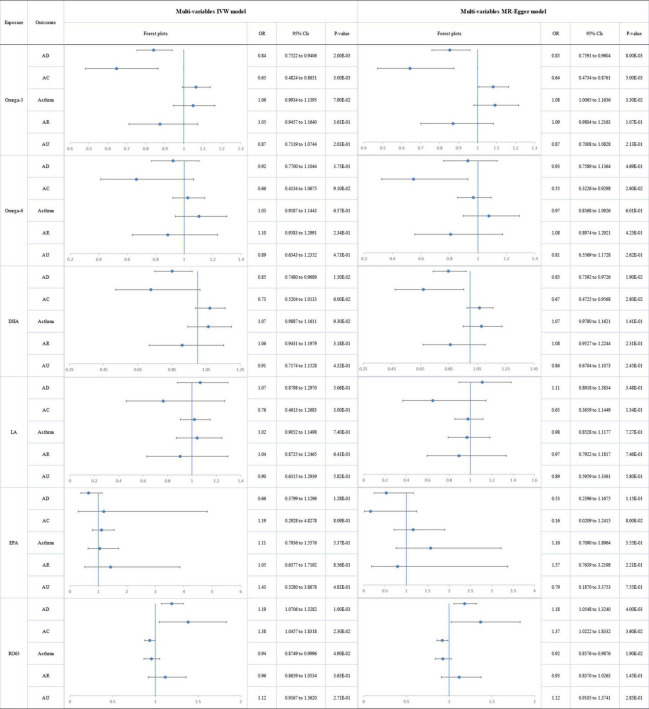
The forest plot of the multivariable Mendelian randomization exploring the associations between genetically determined polyunsaturated fatty acids and allergic diseases adjusted for confounding traits (body mass index, smoking, alcohol intake, age, and income level) OR: odds ratio; CIs: confidence intervals; Omega-3: omega-3 fatty acids; Omega-6: omega-6 fatty acids; DHA: docosahexaenoic acid; LA: linoleic acid; EPA: eicosapentaenoic acid; RO63: ratio of omega-6 fatty acids to omega-3 fatty acids; AD: atopic dermatitis; AC: Atopic conjunctivitis; AR: Allergic rhinitis; AU: Allergic urticaria.

We estimated the independent effects of circulating PUFAs on allergic diseases using multivariable MR conditioned on age, income, BMI, alcohol and smoking (Figure 6) and observed a directly protective effect of omega-3 level on AD (IVW OR_*MVMR*_: 0.841; 95% CI: 0.752 to 0.940; *p* = 2.00E-03) and AC (IVW OR_*MVMR*_: 0.646; 95% CI: 0.482 to 0.865; *p* = 3.00E-03). No significant was observed for omega-6 levels and allergic diseases after adjustment of age, income, BMI, alcohol, and smoking behaviors. Genetic risk of DHA was directly associated with decreased risk of AD (IVW OR_*MVMR*_: 0.851; 95% CI: 0.748 to 0.969; *p* = 1.50E-02). Genetic risk of circulating omega-6:3 was found to have a significant direct association with increased risk of AD (IVW OR_*MVMR*_:1.192; 95% CI:1.071 to 1.328; *p* = 1.00E-03) and AC (IVW OR_*MVMR*_:1.384; 95% CI:1.046 to 1.832; *p* = 2.30E-02). Similarly, there was no significant association of LA and EPA with allergic diseases according to the results of MVMR. Besides, though no significant genetic association was observed between PUFAs and asthma after adjustment of age, income, BMI, alcohol, and smoking behaviors, genetic risk of BMI was found to be associated with a higher risk of asthma. Detailed results of MVMR analyses were presented in Supplementary Tables 7–12.

## Discussion

The current study explored the association between PUFAs and allergic disease risk using both univariable and multivariable two-sample MR. The IVs were used as proxies for PUFAs assessed both as absolute levels and as ratios to produce comparable results. In univariable MR results, Omega-3 levels were found to be likely genetic causal factors associated with decreased risk of some allergic diseases including AD, AC and AU. Genetic predisposition to high omega-6 and DHA levels was suggestively associated with reduced risk of AC. However, the genetic predisposition to high omega-6:3 showed a causal association with an increased risk of AD and AC, and this may constitute a susceptibility factor contributing to the pathogenesis of AD and AC. According to the results of MVMR, the independent protective effect of omega-3 and DHA on AD was identified in our study, as well as omega-3 for AC. Besides, omega-6:3 was independently associated with AD and AC. However, those results for asthma should be interpreted with caution as no specific GWAS data related to allergic asthma could be accessible and used in the present study.

Health benefits of PUFAs have been documented elsewhere and omega-3 (including DHA) have been associated with improvements in cardiovascular health, neurodevelopment and diabetes (52, 53) with omega-6 implicated in hair growth, lipid metabolism, and bone health (54–56). However, omega-3 and omega-6 compete for the same desaturation and elongation enzymes, and an increased ratio of omega-6 to omega-3 may reduce the benefits of omega-3 and increase the probability of inflammatory diseases (57). Inconsistencies have arisen from epidemiological studies, RCTs and meta-analyses into the effects of PUFAs intake during pregnancy (15, 58), biomarker levels (18, 59–64), maternal/individual early life PUFA supplementation, and impacts on the risk of allergic diseases in the offspring or during individual later life (12, 14, 15, 65–67). For the association between PUFAs in plasma and allergic disease, there was evidence showing that higher levels of total omega-3 fatty acid, DHA and EPA in maternal and infant plasma were associated with a lower prevalence of IgE-associated disease (such as eczema) in a dose-dependent manner (68). Reduced concentration of serum omega-3 fatty acids was also identified to characterize women with extensive eczema (69). Previous observational studies mainly provided evidence for the effects of PUFA composition of maternal and umbilical cord plasma on infants or early childhood allergic diseases, while the results in this MR study demonstrated a direct genetic association of circulating PUFAs with allergic skin diseases, especially for the protective effects of omega-3 fatty acid on AD and AC.

Multiple levels of research evidence should be considered when establishing causal effects but observational research under different conditions is susceptible to confounding factors reducing the accuracy of conclusions. Therefore, correlations reported by observational studies cannot be equated with direct causal correlation. MR avoids the influence of confounding factors through genetic instrumental variables and accurate causal assessments may be made. Caution should also be exercised in comparing RCTs with MR effects since genetic susceptibility is considered lifelong, while the effects of dietary supplementation in intervention experiments last only for the duration of the trial. Long-term exposure may be superior, given the long development period of allergic diseases.

The current study is the first MR analysis of PUFAs and allergic diseases and has several advantages. Firstly, compared with the inherent limitations of observational studies, MR studies are less likely to be affected by reverse causality and confounding. Secondly, extensive GWAS sample data, two separate sets of IVs and different methodologies were applied to causal association assessment to improve reliability. Moreover, the causal relationship was extended from single PUFA levels to include the ratio of omega-6 to omega-3. Several limitations must be acknowledged. Firstly, the present study is limited to individuals of European ancestry and may not be generalized to other races. Secondly, inconsistencies in pleiotropy detection and the occurrence of potential driving SNPs are difficult to interpret and may cause bias. Thirdly, the GWAS effect size is based on circulatory PUFA concentration rather than membrane concentration, and membrane association may be more significant given the cell signaling of fatty acid receptors and immune responses (70, 71). Lastly, some more specific and targeted GWAS datasets, such as allergic asthma and eicosapentaenoic acid, were unavailable. However, as genetic instruments continue to improve, MR studies could shed further light on the significance of individual PUFA associations with the risk of specific allergic diseases.

## Conclusion

In conclusion, through both univariable and multivariable MR analyses, our study demonstrated that higher PUFA concentrations (omega-3, DHA) and lower omega-6:3 ratios were associated with a lower risk of some allergic diseases (such as AD and AC). The strongest evidence concerned the protective effect of omega-3. This signifies the substantial clinical value of circulating PUFA levels and omega-6:3 on some allergic diseases and may assist with early diagnosis and enable more efficient targeting for prevention and therapy.

## Data availability statement

The original contributions presented in this study are included in the article/Supplementary material, further inquiries can be directed to the corresponding author/s.

## Author contributions

ZC and JW conceived the study, participated in its design and coordination, and critically revised the manuscript. YL and ZC searched the databases. ZC, QL, and YL reviewed the GWAS datasets and finished the data collection. ZC and YL finished the data analysis. YL drafted the manuscript. YL, JW, and ZC had full access to all the data collection, analysis, and interpretation. All authors read and approved the final manuscript.

## References

[1] Brunner PM Silverberg JI Guttman-Yassky E Paller AS Kabashima K Amagai M Increasing comorbidities suggest that atopic dermatitis is a systemic disorder. *J Invest Dermatol.* (2017) 137:18–25. 10.1016/j.jid.2016.08.022 27771048

[2] Bantz SK Zhu Z Zheng T. The atopic march: progression from atopic dermatitis to allergic rhinitis and asthma. *J Clin Cell Immunol.* (2014) 5:202. 10.4172/2155-9899.1000202 PMC4240310 25419479

[3] Spergel. Atopic dermatitis and the atopic march. *J Allergy Clin Immunol.* (2003) 112:S118–27. 14657842 10.1016/j.jaci.2003.09.033

[4] Pawankar R Caonica GW Holgate ST *World Allergy Organization (WAO) White Book On Allergy 2011-2012: Executive Summary[J]. Japanese Journal Of Allergology.* (2011). Available online at: http://www.worldallergy.org

[5] Pawankar R Holgate ST Canonica GW Lockey RF Blaiss MS *World Allergy Organization (WAO) White Book on Allergy*. Milwaukee, WI: World Allergy Organization (2013).

[6] Spergel JM. Epidemiology of atopic dermatitis and atopic march in children. *Immunol Allergy Clin North Am.* (2010) 30:269–80. 10.1016/j.iac.2010.06.003 20670812

[7] Muraro A Dubois AE DunnGalvin A Hourihane JO de Jong NW Meyer R EAACI food allergy and anaphylaxis guidelines. Food allergy health-related quality of life measures. *Allergy.* (2014) 69:845–53. 10.1111/all.12405 24785644

[8] Cook Q Argenio K Lovinsky-Desir S. The impact of environmental injustice and social determinants of health on the role of air pollution in asthma and allergic disease in the United States. *J Allergy Clin Immunol.* (2021) 148:1089.e–101.e. 10.1016/j.jaci.2021.09.018 34743831

[9] Ferreira MA Vonk JM Baurecht H Marenholz I Tian C Hoffman JD Shared genetic origin of asthma, hay fever and eczema elucidates allergic disease biology. *Nat Genet.* (2017) 49:1752–7. 10.1038/ng.3985 29083406 PMC5989923

[10] Calder PC Krauss-Etschmann S de Jong EC Dupont C Frick JS Frokiaer H Early nutrition and immunity - progress and perspectives. *Br J Nutr.* (2006) 96:774–90. 17010239

[11] Clausen M Jonasson K Keil T Beyer K Sigurdardottir ST. Fish oil in infancy protects against food allergy in Iceland-results from a birth cohort study. *Allergy.* (2018) 73:1305–12. 10.1111/all.13385 29318622 PMC6032905

[12] Hansen S Strøm M Maslova E Dahl R Hoffmann HJ Rytter D Fish oil supplementation during pregnancy and allergic respiratory disease in the adult offspring. *J Allergy Clin Immunol.* (2017) 139:104.e–11.e. 10.1016/j.jaci.2016.02.042 27246522

[13] Kumar A Mastana SS Lindley MR. n-3 Fatty acids and asthma. *Nutr Res Rev.* (2016) 29:1–16. 10.1017/s0954422415000116 26809946

[14] Anandan C Nurmatov U Sheikh A. Omega 3 and 6 oils for primary prevention of allergic disease: systematic review and meta-analysis. *Allergy.* (2009) 64:840–8. 10.1111/j.1398-9995.2009.02042.x 19392990

[15] Best KP Gold M Kennedy D Martin J Makrides M. Omega-3 long-chain PUFA intake during pregnancy and allergic disease outcomes in the offspring: a systematic review and meta-analysis of observational studies and randomized controlled trials. *Am J Clin Nutr.* (2016) 103:128–43. 10.3945/ajcn.115.111104 26675770

[16] Reichardt P Müller D Posselt U Vorberg B Diez U Schlink U Fatty acids in colostrum from mothers of children at high risk of atopy in relation to clinical and laboratory signs of allergy in the first year of life. *Allergy.* (2004) 59:394–400. 10.1111/j.1398-9995.2003.00429.x 15005762

[17] Rosenlund H Fagerstedt S Alm J Mie A. Breastmilk fatty acids in relation to sensitization - the ALADDIN birth cohort. *Allergy.* (2016) 71:1444–52. 10.1111/all.12896 27043329

[18] Yu YM Chan YH Calder PC Hardjojo A Soh SE Lim AL Maternal PUFA status and offspring allergic diseases up to the age of 18 months. *Br J Nutr.* (2015) 113:975–83. 10.1017/s000711451500001x 25746049

[19] Simopoulos AP Fatty Acids | Omega-3 polyunsaturated. In: Caballero B Editor. *Encyclopedia of Human Nutrition*, 2nd Edn. Oxford: Elsevier (2005). p. 205–19. 10.1016/B0-12-226694-3/00120-4

[20] Zhang GQ Liu B Li J Luo CQ Zhang Q Chen JL Fish intake during pregnancy or infancy and allergic outcomes in children: a systematic review and meta-analysis. *Pediatr Allergy Immunol.* (2017) 28:152–61. 10.1111/pai.12648 27590571

[21] Wang P Liu L Lei SF. Causal effects of homocysteine levels on the changes of bone mineral density and risk for bone fracture: a two-sample mendelian randomization study. *Clin Nutr.* (2021) 40:1588–95. 10.1016/j.clnu.2021.02.045 33744603

[22] Bennett DA Holmes MV. Mendelian randomisation in cardiovascular research: an introduction for clinicians. *Heart.* (2017) 103:1400–7. 10.1136/heartjnl-2016-310605 28596306 PMC5574403

[23] Liao LZ Zhang SZ Li WD Liu Y Li JP Zhuang XD Serum albumin and atrial fibrillation: insights from epidemiological and mendelian randomization studies. *Eur J Epidemiol.* (2020) 35:113–22. 10.1007/s10654-019-00583-6 31741136

[24] Evans DM Davey Smith G. Mendelian randomization: new applications in the coming age of hypothesis-free causality. *Annu Rev Genomics Hum Genet.* (2015) 16:327–50. 10.1146/annurev-genom-090314-050016 25939054

[25] Pierce BL Burgess S. Efficient design for mendelian randomization studies: subsample and 2-sample instrumental variable estimators. *Am J Epidemiol.* (2013) 178:1177–84. 10.1093/aje/kwt084 23863760 PMC3783091

[26] Burgess S Thompson SG. Multivariable mendelian randomization: the use of pleiotropic genetic variants to estimate causal effects. *Am J Epidemiol.* (2015) 181:251–60. 10.1093/aje/kwu283 25632051 PMC4325677

[27] Burgess S Scott RA Timpson NJ Davey Smith G Thompson SG. Using published data in mendelian randomization: a blueprint for efficient identification of causal risk factors. *Eur J Epidemiol.* (2015) 30:543–52. 10.1007/s10654-015-0011-z 25773750 PMC4516908

[28] Davies NM Holmes MV Davey Smith G. Reading mendelian randomisation studies: a guide, glossary, and checklist for clinicians. *BMJ.* (2018) 362:k601. 10.1136/bmj.k601 30002074 PMC6041728

[29] Julkunen H Cichońska A Slagboom PE Würtz P. Metabolic biomarker profiling for identification of susceptibility to severe pneumonia and COVID-19 in the general population. *Elife.* (2021) 10:e63033. 10.7554/eLife.63033 33942721 PMC8172246

[30] Soininen P Kangas AJ Würtz P Suna T Ala-Korpela M. Quantitative serum nuclear magnetic resonance metabolomics in cardiovascular epidemiology and genetics. *Circ Cardiovasc Genet.* (2015) 8:192–206. 10.1161/circgenetics.114.000216 25691689

[31] Soininen P Kangas AJ Würtz P Tukiainen T Tynkkynen T Laatikainen R High-throughput serum NMR metabonomics for cost-effective holistic studies on systemic metabolism. *Analyst.* (2009) 134:1781–5. 10.1039/b910205a 19684899

[32] Würtz P Kangas AJ Soininen P Lawlor DA Davey Smith G Ala-Korpela M. Quantitative serum nuclear magnetic resonance metabolomics in large-scale epidemiology: a primer on -omic technologies. *Am J Epidemiol.* (2017) 186:1084–96. 10.1093/aje/kwx016 29106475 PMC5860146

[33] Shin SY Fauman EB Petersen AK Krumsiek J Santos R Huang J An atlas of genetic influences on human blood metabolites. *Nat Genet.* (2014) 46:543–50. 10.1038/ng.2982 24816252 PMC4064254

[34] Krumsiek J Suhre K Evans AM Mitchell MW Mohney RP Milburn MV Mining the unknown: a systems approach to metabolite identification combining genetic and metabolic information. *PLoS Genet.* (2012) 8:e1003005. 10.1371/journal.pgen.1003005 23093944 PMC3475673

[35] Suhre K Shin SY Petersen AK Mohney RP Meredith D Wägele B Human metabolic individuality in biomedical and pharmaceutical research. *Nature.* (2011) 477:54–60. 10.1038/nature10354 21886157 PMC3832838

[36] Bowden J Del Greco MF Minelli C Davey Smith G Sheehan NA Thompson JR. Assessing the suitability of summary data for two-sample mendelian randomization analyses using MR-Egger regression: the role of the I2 statistic. *Int J Epidemiol.* (2016) 45:1961–74. 10.1093/ije/dyw220 27616674 PMC5446088

[37] Sanderson E Windmeijer F. A weak instrument [Formula: see text]-test in linear IV models with multiple endogenous variables. *J Econo.* (2016) 190:212–21. 10.1016/j.jeconom.2015.06.004 29129953 PMC5669336

[38] Burgess S Butterworth A Thompson SG. Mendelian randomization analysis with multiple genetic variants using summarized data. *Genet Epidemiol.* (2013) 37:658–65. 10.1002/gepi.21758 24114802 PMC4377079

[39] Staley JR Burgess S. Semiparametric methods for estimation of a nonlinear exposure-outcome relationship using instrumental variables with application to Mendelian randomization. *Genet Epidemiol.* (2017) 41:341–52. 10.1002/gepi.22041 28317167 PMC5400068

[40] Bowden J Davey Smith G Burgess S. Mendelian randomization with invalid instruments: effect estimation and bias detection through egger regression. *Int J Epidemiol.* (2015) 44:512–25. 10.1093/ije/dyv080 26050253 PMC4469799

[41] Bowden J Davey Smith G Haycock PC Burgess S. Consistent estimation in mendelian randomization with some invalid instruments using a weighted median estimator. *Genet Epidemiol.* (2016) 40:304–14. 10.1002/gepi.21965 27061298 PMC4849733

[42] Hartwig FP Davey Smith G Bowden J. Robust inference in summary data mendelian randomization via the zero modal pleiotropy assumption. *Int J Epidemiol.* (2017) 46:1985–98. 10.1093/ije/dyx102 29040600 PMC5837715

[43] Zhao Q Wang J Hemani G Bowden J Small DS *Statistical Inference In Two-Sample Summary-Data Mendelian Randomization Using Robust Adjusted Profile Score. arXiv: 1801.09652*. (2018). Available online at: https://ui.adsabs.harvard.edu/abs/2018arXiv180109652Z (accessed January 1, 2018).

[44] Verbanck M Chen CY Neale B Do R. Detection of widespread horizontal pleiotropy in causal relationships inferred from mendelian randomization between complex traits and diseases. *Nat Genet.* (2018) 50:693–8. 10.1038/s41588-018-0099-7 29686387 PMC6083837

[45] Greco MF Minelli C Sheehan NA Thompson JR. Detecting pleiotropy in mendelian randomisation studies with summary data and a continuous outcome. *Stat Med.* (2015) 34:2926–40. 10.1002/sim.6522 25950993

[46] Burgess S Thompson SG. Interpreting findings from mendelian randomization using the MR-Egger method. *Eur J Epidemiol.* (2017) 32:377–89. 10.1007/s10654-017-0255-x 28527048 PMC5506233

[47] Slob EAW Burgess S. A comparison of robust mendelian randomization methods using summary data. *Genet Epidemiol.* (2020) 44:313–29. 10.1002/gepi.22295 32249995 PMC7317850

[48] Sanderson E Davey Smith G Windmeijer F Bowden J. An examination of multivariable mendelian randomization in the single-sample and two-sample summary data settings. *Int J Epidemiol.* (2019) 48:713–27. 10.1093/ije/dyy262 30535378 PMC6734942

[49] Rees JMB Wood AM Burgess S. Extending the MR-egger method for multivariable mendelian randomization to correct for both measured and unmeasured pleiotropy. *Stat Med.* (2017) 36:4705–18. 10.1002/sim.7492 28960498 PMC5725762

[50] Yavorska OO Burgess S. Mendelian randomization: an R package for performing mendelian randomization analyses using summarized data. *Int J Epidemiol.* (2017) 46:1734–9. 10.1093/ije/dyx034 28398548 PMC5510723

[51] Skrivankova VW Richmond RC Woolf BAR Yarmolinsky J Davies NM Swanson SA Strengthening the reporting of observational studies in epidemiology using mendelian randomization: the STROBE-MR statement. *JAMA.* (2021) 326:1614–21. 10.1001/jama.2021.18236 34698778

[52] Shahidi F Ambigaipalan P. Omega-3 polyunsaturated fatty acids and their health benefits. *Annu Rev Food Sci Technol.* (2018) 9:345–81. 10.1146/annurev-food-111317-095850 29350557

[53] Elagizi A Lavie CJ O’Keefe E Marshall K O’Keefe JH Milani RV. An update on omega-3 polyunsaturated fatty acids and cardiovascular health. *Nutrients.* (2021) 13:204. 10.3390/nu13010204 33445534 PMC7827286

[54] Le Floc’h C Cheniti A Connétable S Piccardi N Vincenzi C Tosti A. Effect of a nutritional supplement on hair loss in women. *J Cosmet Dermatol.* (2015) 14:76–82. 10.1111/jocd.12127 25573272

[55] Bjørklund G Dadar M Doşa MD Chirumbolo S Pen JJ. Insights into the effects of dietary omega-6/omega-3 polyunsaturated fatty acid (PUFA) ratio on oxidative metabolic pathways of oncological bone disease and global health. *Curr Med Chem.* (2021) 28:1672–82. 10.2174/0929867327666200427095331 32338204

[56] Hooper L Al-Khudairy L Abdelhamid AS Rees K Brainard JS Brown TJ Omega-6 fats for the primary and secondary prevention of cardiovascular disease. *Cochrane Database Syst Rev.* (2018) 7:Cd011094. 10.1002/14651858.CD011094.pub3 30019765 PMC6513455

[57] Klemens CM Berman DR Mozurkewich EL. The effect of perinatal omega-3 fatty acid supplementation on inflammatory markers and allergic diseases: a systematic review. *Bjog.* (2011) 118:916–25. 10.1111/j.1471-0528.2010.02846.x 21658192

[58] Venter C Agostoni C Arshad SH Ben-Abdallah M Du Toit G Fleischer DM Dietary factors during pregnancy and atopic outcomes in childhood: a systematic review from the European academy of allergy and clinical immunology. *Pediatr Allergy Immunol.* (2020) 31:889–912. 10.1111/pai.13303 32524677 PMC9588404

[59] Standl M Demmelmair H Koletzko B Heinrich J. Cord blood LC-PUFA composition and allergic diseases during the first 10 yr. Results from the LISAplus study. *Pediatr Allergy Immunol.* (2014) 25:344–50. 10.1111/pai.12212 24576150 PMC4238817

[60] Barman M Johansson S Hesselmar B Wold AE Sandberg AS Sandin A. High levels of both n-3 and n-6 long-chain polyunsaturated fatty acids in cord serum phospholipids predict allergy development. *PLoS One.* (2013) 8:e67920. 10.1371/journal.pone.0067920 23874467 PMC3707846

[61] Rucci E den Dekker HT de Jongste JC Steenweg-de-Graaff J Gaillard R Pasmans SG Maternal fatty acid levels during pregnancy, childhood lung function and atopic diseases. The Generation R Study. *Clin Exp Allergy.* (2016) 46:461–71. 10.1111/cea.12613 26285050

[62] Notenboom ML Mommers M Jansen EH Penders J Thijs C. Maternal fatty acid status in pregnancy and childhood atopic manifestations: KOALA birth cohort study. *Clin Exp Allergy.* (2011) 41:407–16. 10.1111/j.1365-2222.2010.03672.x 21255139

[63] Pike KC Calder PC Inskip HM Robinson SM Roberts GC Cooper C Maternal plasma phosphatidylcholine fatty acids and atopy and wheeze in the offspring at age of 6 years. *Clin Dev Immunol.* (2012) 2012:474613. 10.1155/2012/474613 23049600 PMC3463812

[64] Newson RB Shaheen SO Henderson AJ Emmett PM Sherriff A Calder PC. Umbilical cord and maternal blood red cell fatty acids and early childhood wheezing and eczema. *J Allergy Clin Immunol.* (2004) 114:531–7. 10.1016/j.jaci.2004.05.010 15356553

[65] Zhang Y Lin J Zhou R Zheng X Dai J. Effect of omega-3 fatty acids supplementation during childhood in preventing allergic disease: a systematic review and meta-analysis. *J Asthma.* (2021) 58:523–36. 31880179 10.1080/02770903.2019.1709866

[66] Best KP Sullivan T Palmer D Gold M Kennedy DJ Martin J Prenatal fish oil supplementation and allergy: 6-year follow-up of a randomized controlled trial. *Pediatrics.* (2016) 137:e20154443. 10.1542/peds.2015-4443 27225316

[67] Vahdaninia M Mackenzie H Dean T Helps S. ω-3 LCPUFA supplementation during pregnancy and risk of allergic outcomes or sensitization in offspring: a systematic review and meta-analysis. *Ann Allergy Asthma Immunol.* (2019) 122:302.e–13.e. 10.1016/j.anai.2018.12.008 30552987

[68] Furuhjelm C Warstedt K Fagerås M Fälth-Magnusson K Larsson J Fredriksson M Allergic disease in infants up to 2 years of age in relation to plasma omega-3 fatty acids and maternal fish oil supplementation in pregnancy and lactation. *Pediatr Allergy Immunol.* (2011) 22:505–14. 10.1111/j.1399-3038.2010.01096.x 21332799

[69] Johansson S Wold AE Sandberg AS. Low breast milk levels of long-chain n-3 fatty acids in allergic women, despite frequent fish intake. *Clin Exp Allergy.* (2011) 41:505–15. 10.1111/j.1365-2222.2010.03678.x 21338426 PMC3085074

[70] Son SE Koh JM Im DS. Activation of free fatty acid receptor 4 (FFA4) ameliorates ovalbumin-induced allergic asthma by suppressing activation of dendritic and mast cells in mice. *Int J Mol Sci.* (2022) 23:5270. 10.3390/ijms23095270 35563671 PMC9100770

[71] Patel D Newell M Goruk S Richard C Field CJ. Long chain polyunsaturated fatty acids docosahexaenoic acid and arachidonic acid supplementation in the suckling and the post-weaning diet influences the immune system development of T helper type-2 bias brown norway rat offspring. *Front Nutr.* (2021) 8:769293. 10.3389/fnut.2021.769293 34790691 PMC8592062

