# Supplementary material for: The causal association of polyunsaturated fatty acids with allergic disease: A two-sample Mendelian randomization study
Source: Front Nutr. 2022 Sep 9;9:962787. doi: 10.3389/fnut.2022.962787 (PMC9500587; doi:10.3389/fnut.2022.962787)
Supplement: Supplementary file 1 [file Data_Sheet_1.docx]

**Supplementary Figure 1.** Step-by-step flow chart of analytical methods used in this MR analysis.

**Supplementary Figure 2.** Forest plot of univariable Mendelian randomisation analyses exploring associations between linoleic acid to allergic diseases risk using different Mendelian randomization statistical models. OR: odds ratio; CIs: confidence intervals.

**Supplementary Figure 3.** Forest plot of univariable Mendelian randomisation analyses exploring associations between eicosapentaenoic acid to allergic diseases risk using different Mendelian randomization statistical models. OR: odds ratio; CIs: confidence intervals.

**Supplementary Figure 4.** Scatter plots of causal estimates of exposure (Omega-3 fatty acids) on outcomes. The slope of each line corresponding to the estimated MR effect in different models, including the conventional IVW, MBE, WMM, MR-Egger, MR-RAPS and MR-PRESSO methods. The effect of A: AD; B: AC; C: Asthma; D: AR; E: AU. AD: atopic dermatitis; AC: Atopic conjunctivitis; AR: Allergic rhinitis; AU: Allergic urticaria.

**Supplementary Figure 5.** Scatter plots of causal estimates of exposure (Omega-6 fatty acids) on outcomes. The slope of each line corresponding to the estimated MR effect in different models, including the conventional IVW, MBE, WMM, MR-Egger, MR-RAPS and MR-PRESSO methods. The effect of A: AD; B: AC; C: Asthma; D: AR; E: AU. AD: atopic dermatitis; AC: Atopic conjunctivitis; AR: Allergic rhinitis; AU: Allergic urticaria.

**Supplementary Figure 6.** Scatter plots of causal estimates of exposure (Docosahexaenoic acid) on outcomes. The slope of each line corresponding to the estimated MR effect in different models, including the conventional IVW, MBE, WMM, MR-Egger, MR-RAPS and MR-PRESSO methods. The effect of A: AD; B: AC; C: Asthma; D: AR; E: AU. AD: atopic dermatitis; AC: Atopic conjunctivitis; AR: Allergic rhinitis; AU: Allergic urticaria.

**Supplementary Figure 7.** Scatter plots of causal estimates of exposure (Ratio of omega-6 fatty acids to omega-3 fatty acids) on outcomes. The slope of each line corresponding to the estimated MR effect in different models, including the conventional IVW, MBE, WMM, MR-Egger, MR-RAPS and MR-PRESSO methods. The effect of A: AD; B: AC; C: Asthma; D: AR; E: AU. AD: atopic dermatitis; AC: Atopic conjunctivitis; AR: Allergic rhinitis; AU: Allergic urticaria.

**Supplementary Figure 8.** Scatter plots of causal estimates of exposure (Linoleic acid) on outcomes. The slope of each line corresponding to the estimated MR effect in different models, including the conventional IVW, MBE, WMM, MR-Egger, MR-RAPS and MR-PRESSO methods. The effect of A: AD; B: AC; C: Asthma; D: AR; E: AU. AD: atopic dermatitis; AC: Atopic conjunctivitis; AR: Allergic rhinitis; AU: Allergic urticaria.

**Supplementary Figure 9.** Scatter plots of causal estimates of exposure (Eicosapentaenoic acid) on outcomes. The slope of each line corresponding to the estimated MR effect in different models, including the conventional IVW, MBE, WMM, MR-Egger, MR-RAPS and MR-PRESSO methods. The effect of A: AD; B: AC; C: Asthma; D: AR; E: AU. AD: atopic dermatitis; AC: Atopic conjunctivitis; AR: Allergic rhinitis; AU: Allergic urticaria.

**Supplementary Figure 10.** Leave-one-out stability tests causal estimates of exposure (Omega-3 fatty acids) on outcomes. Calculate the MR results of the remaining IVs after removing the IVs one by one. The effect of A: AD; B: AC; C: Asthma; D: AR; E: AU. AD: atopic dermatitis; AC: Atopic conjunctivitis; AR: Allergic rhinitis; AU: Allergic urticaria.

**Supplementary Figure 11.** Leave-one-out stability tests causal estimates of exposure (Omega-6 fatty acids) on outcomes. Calculate the MR results of the remaining IVs after removing the IVs one by one. The effect of A: AD; B: AC; C: Asthma; D: AR; E: AU. AD: atopic dermatitis; AC: Atopic conjunctivitis; AR: Allergic rhinitis; AU: Allergic urticaria.

**Supplementary Figure 12.** Leave-one-out stability tests causal estimates of exposure (Docosahexaenoic acid) on outcomes. Calculate the MR results of the remaining IVs after removing the IVs one by one. The effect of A: AD; B: AC; C: Asthma; D: AR; E: AU. AD: atopic dermatitis; AC: Atopic conjunctivitis; AR: Allergic rhinitis; AU: Allergic urticaria.

**Supplementary Figure 13.** Leave-one-out stability tests causal estimates of exposure (Ratio of omega-6 fatty acids to omega-3 fatty acids) on outcomes. Calculate the MR results of the remaining IVs after removing the IVs one by one. The effect of A: AD; B: AC; C: Asthma; D: AR; E: AU. AD: atopic dermatitis; AC: Atopic conjunctivitis; AR: Allergic rhinitis; AU: Allergic urticaria.

**Supplementary Figure 14.** Leave-one-out stability tests causal estimates of exposure (Linoleic acid) on outcomes. Calculate the MR results of the remaining IVs after removing the IVs one by one. The effect of A: AD; B: AC; C: Asthma; D: AR; E: AU. AD: atopic dermatitis; AC: Atopic conjunctivitis; AR: Allergic rhinitis; AU: Allergic urticaria.

**Supplementary Figure 15.** Leave-one-out stability tests causal estimates of exposure (Eicosapentaenoic acid) on outcomes. Calculate the MR results of the remaining IVs after removing the IVs one by one. The effect of A: AD; B: AC; C: Asthma; D: AR; E: AU. AD: atopic dermatitis; AC: Atopic conjunctivitis; AR: Allergic rhinitis; AU: Allergic urticaria.

**
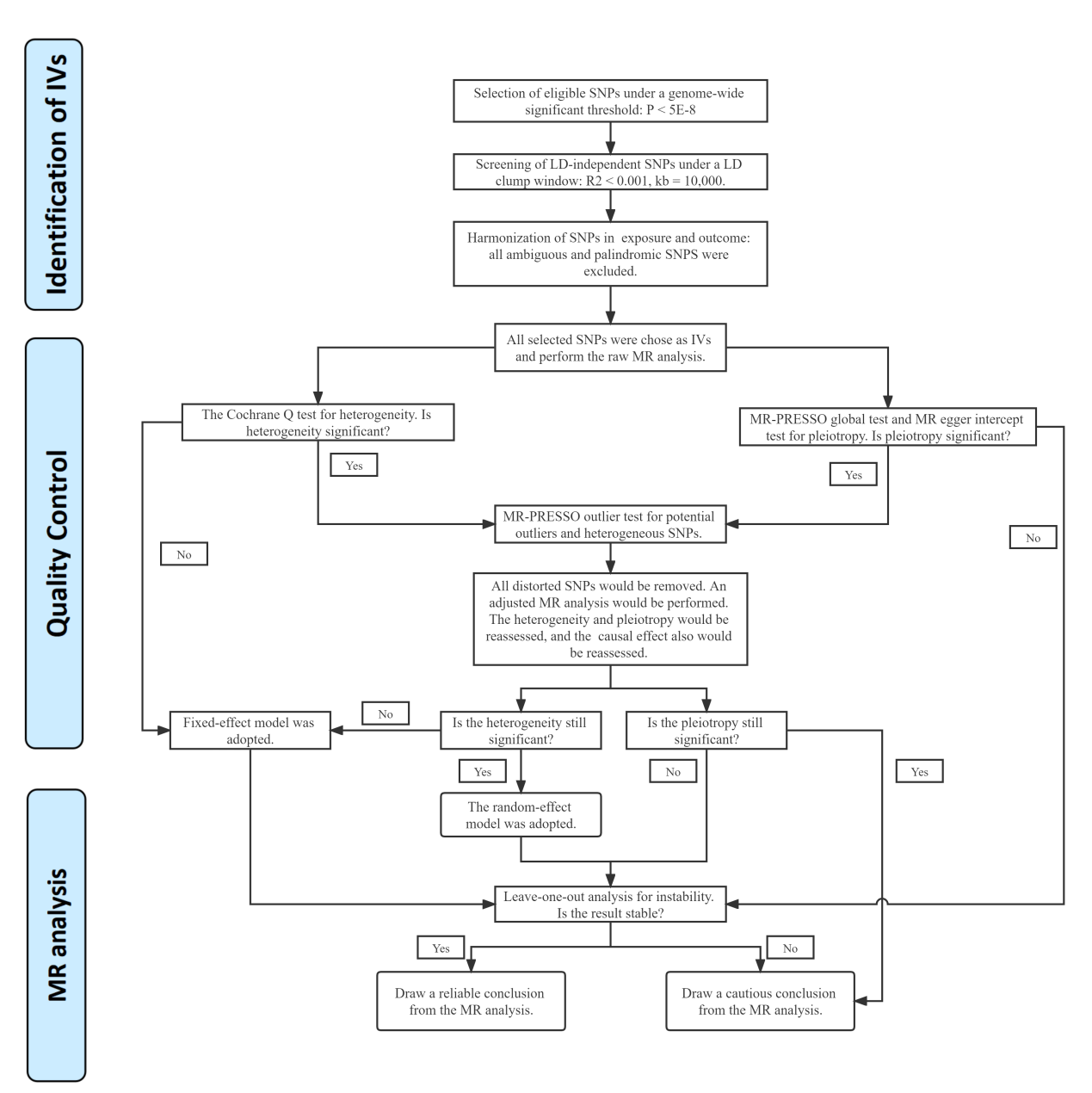
**

**Supplementary Figure 1.** Step-by-step flow chart of analytical methods used in this MR analysis.


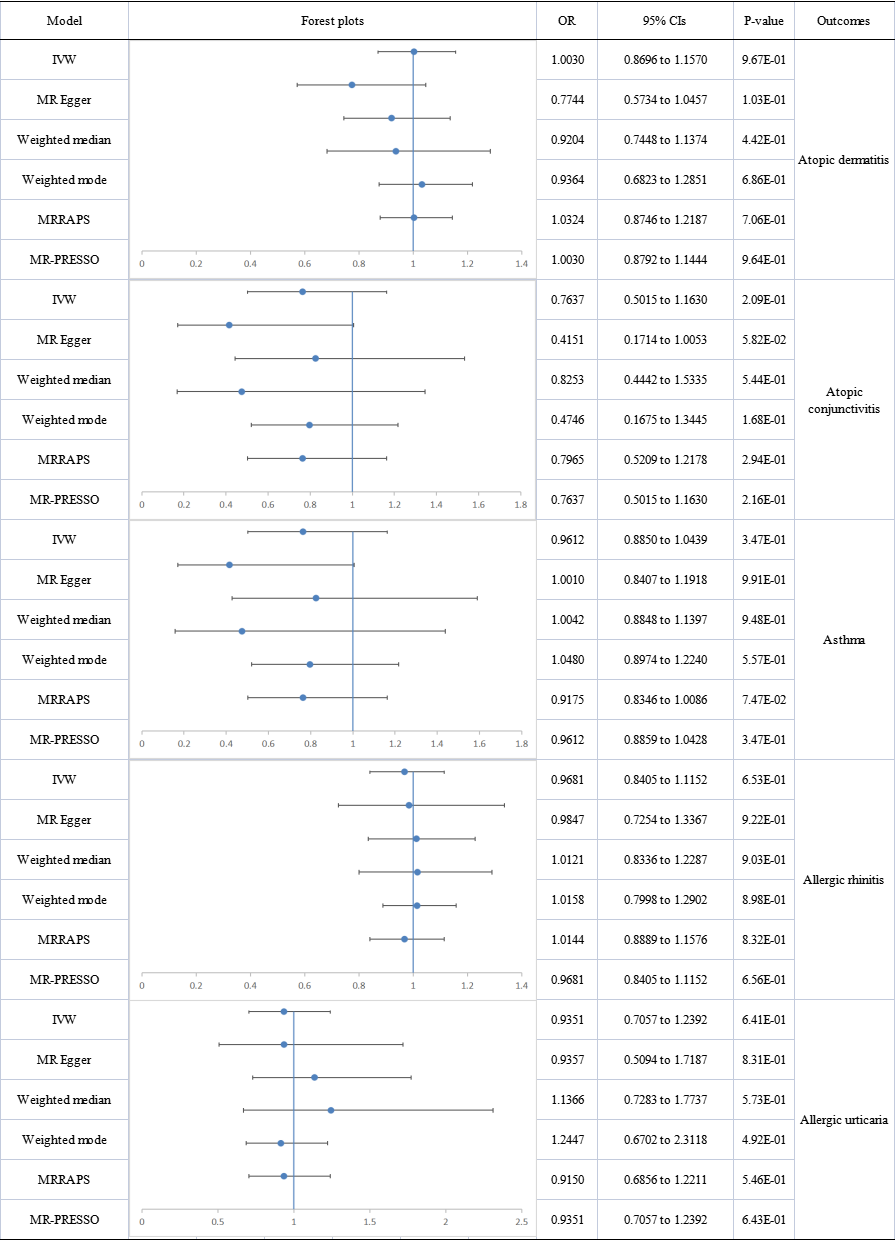


**Supplementary Figure 2.** Forest plot of univariable Mendelian randomisation analyses exploring associations between linoleic acid to allergic diseases risk using different Mendelian randomization statistical models. OR: odds ratio; CIs: confidence intervals.


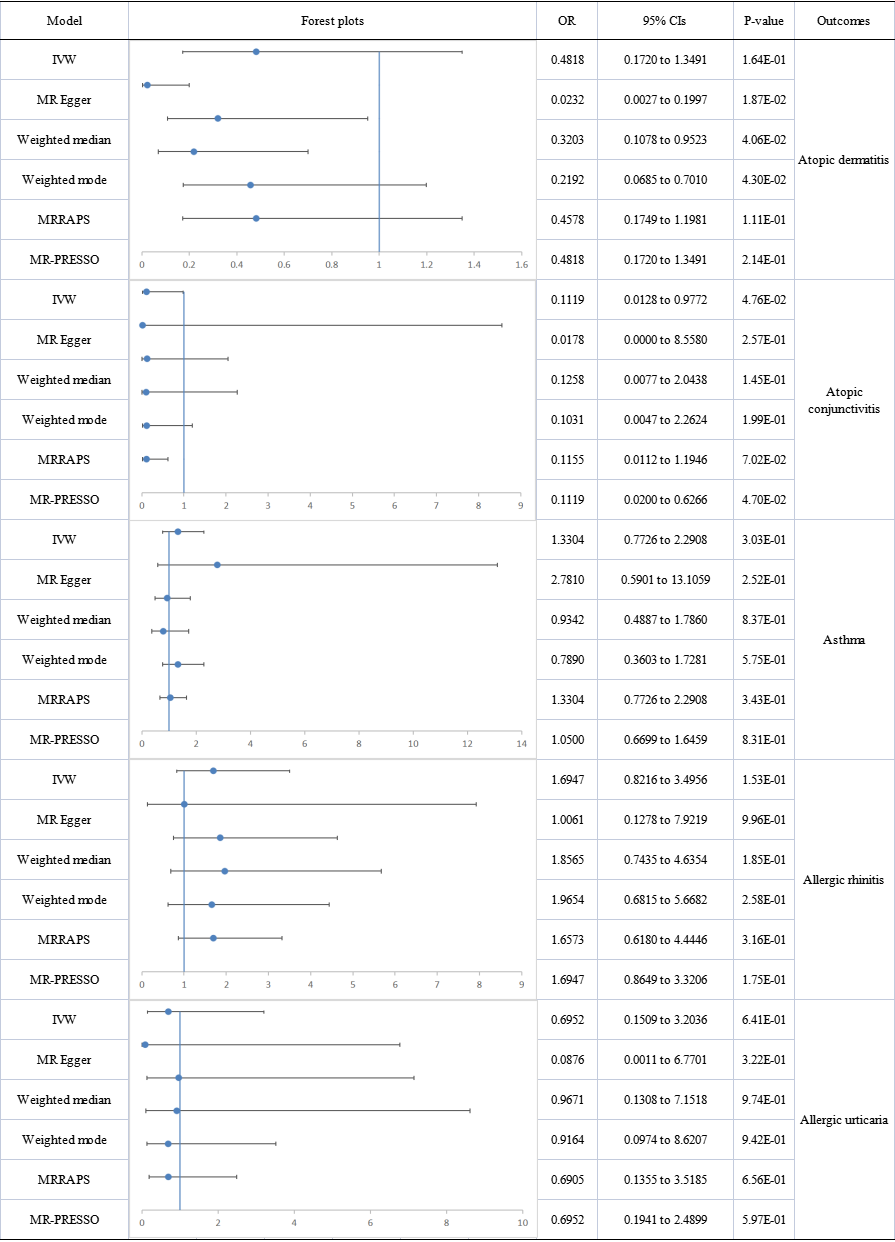


**Supplementary Figure 3.** Forest plot of univariable Mendelian randomisation analyses exploring associations between eicosapentaenoic acid to allergic diseases risk using different Mendelian randomization statistical models. OR: odds ratio; CIs: confidence intervals.


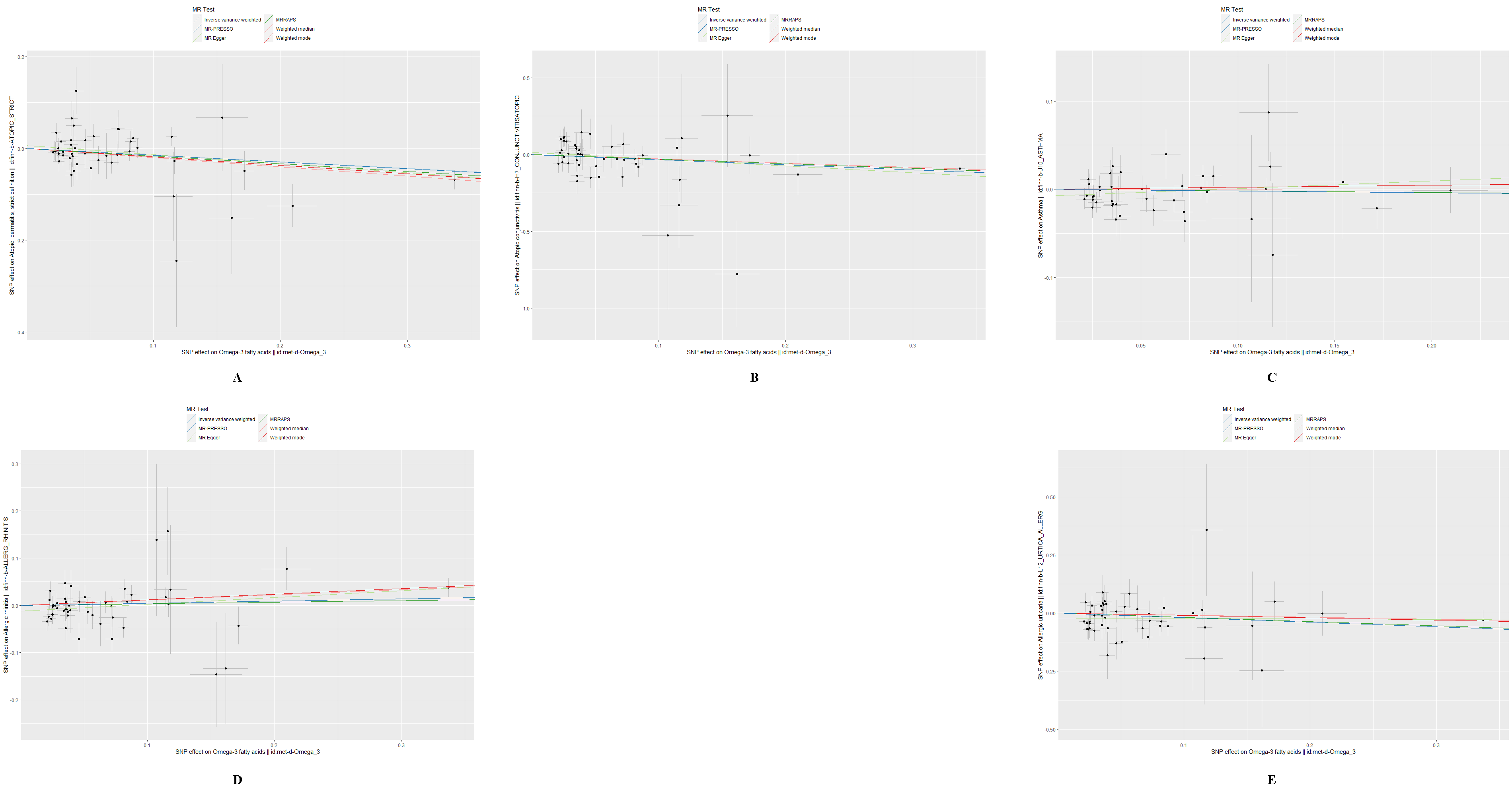


**Supplementary Figure 4.** Scatter plots of causal estimates of exposure (Omega-3 fatty acids) on outcomes. The slope of each line corresponding to the estimated MR effect in different models, including the conventional IVW, MBE, WMM, MR-Egger, MR-RAPS and MR-PRESSO methods. The effect of A: AD; B: AC; C: Asthma; D: AR; E: AU. AD: atopic dermatitis; AC: Atopic conjunctivitis; AR: Allergic rhinitis; AU: Allergic urticaria.


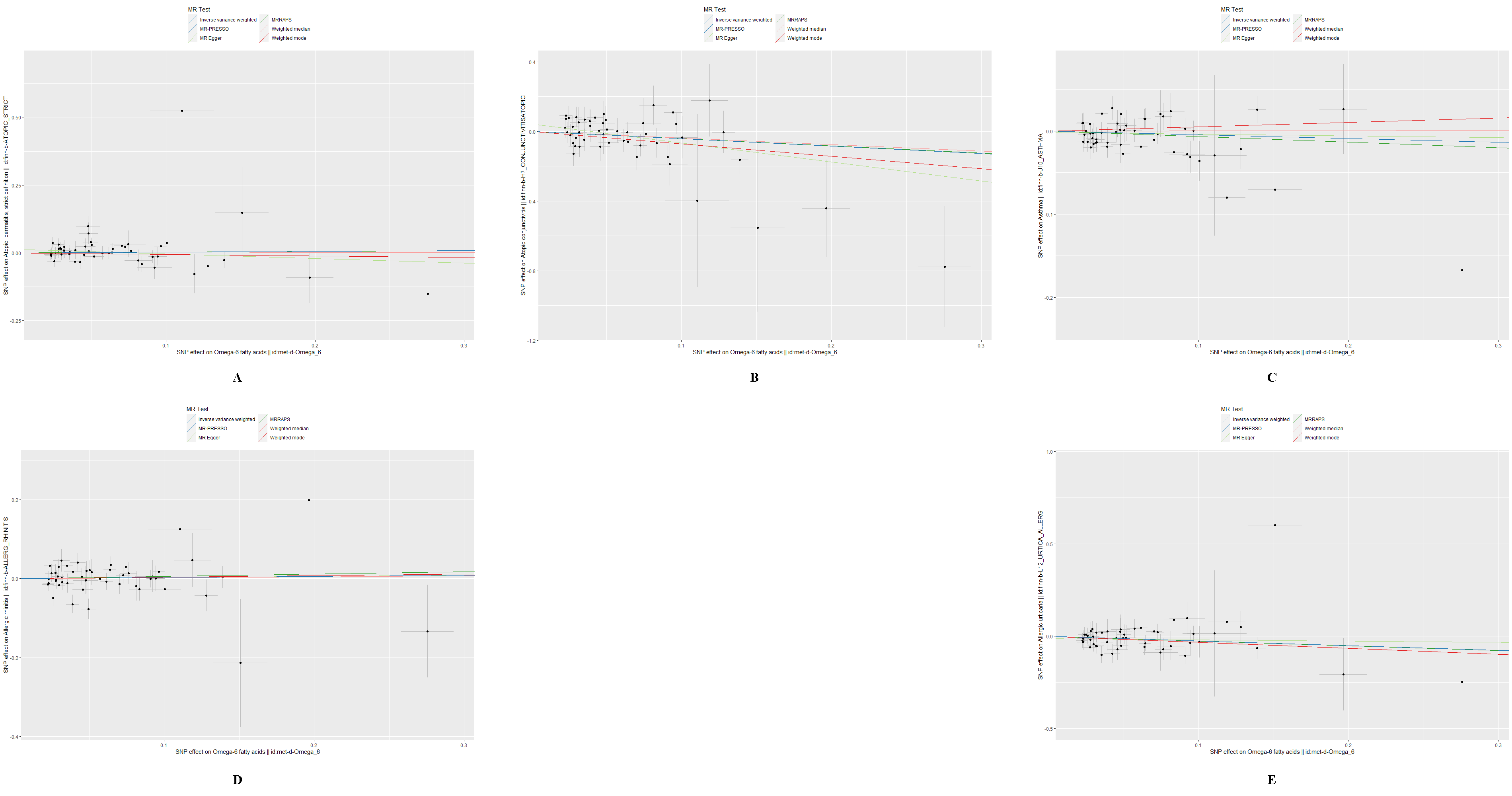


**Supplementary Figure 5.** Scatter plots of causal estimates of exposure (Omega-6 fatty acids) on outcomes. The slope of each line corresponding to the estimated MR effect in different models, including the conventional IVW, MBE, WMM, MR-Egger, MR-RAPS and MR-PRESSO methods. The effect of A: AD; B: AC; C: Asthma; D: AR; E: AU. AD: atopic dermatitis; AC: Atopic conjunctivitis; AR: Allergic rhinitis; AU: AU: Allergic urticaria.


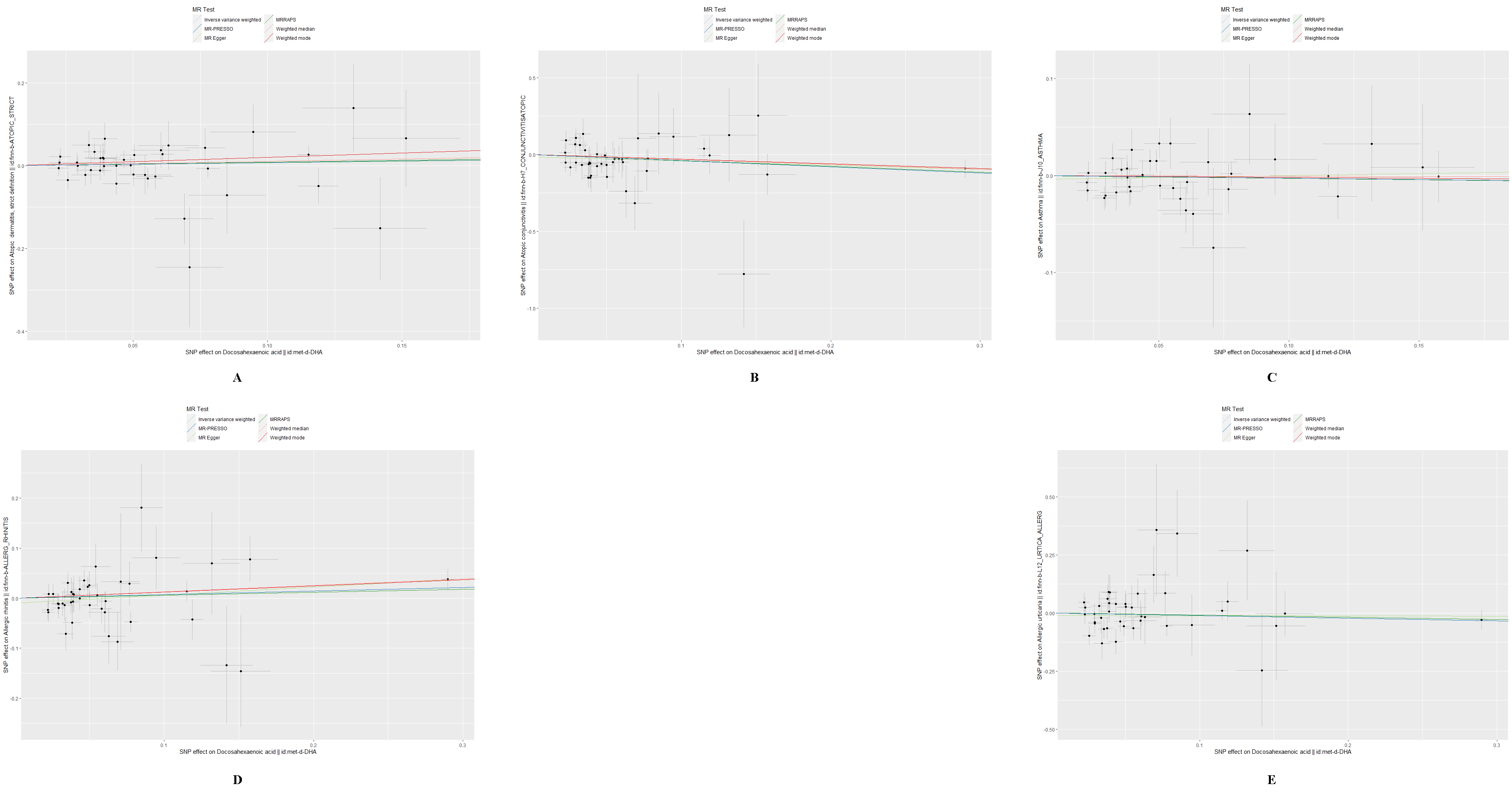


**Supplementary Figure 6.** Scatter plots of causal estimates of exposure (Docosahexaenoic acid) on outcomes. The slope of each line corresponding to the estimated MR effect in different models, including the conventional IVW, MBE, WMM, MR-Egger, MR-RAPS and MR-PRESSO methods. The effect of A: AD; B: AC; C: Asthma; D: AR; E: AU. AD: atopic dermatitis; AC: Atopic conjunctivitis; AR: Allergic rhinitis; AU: Allergic urticaria.


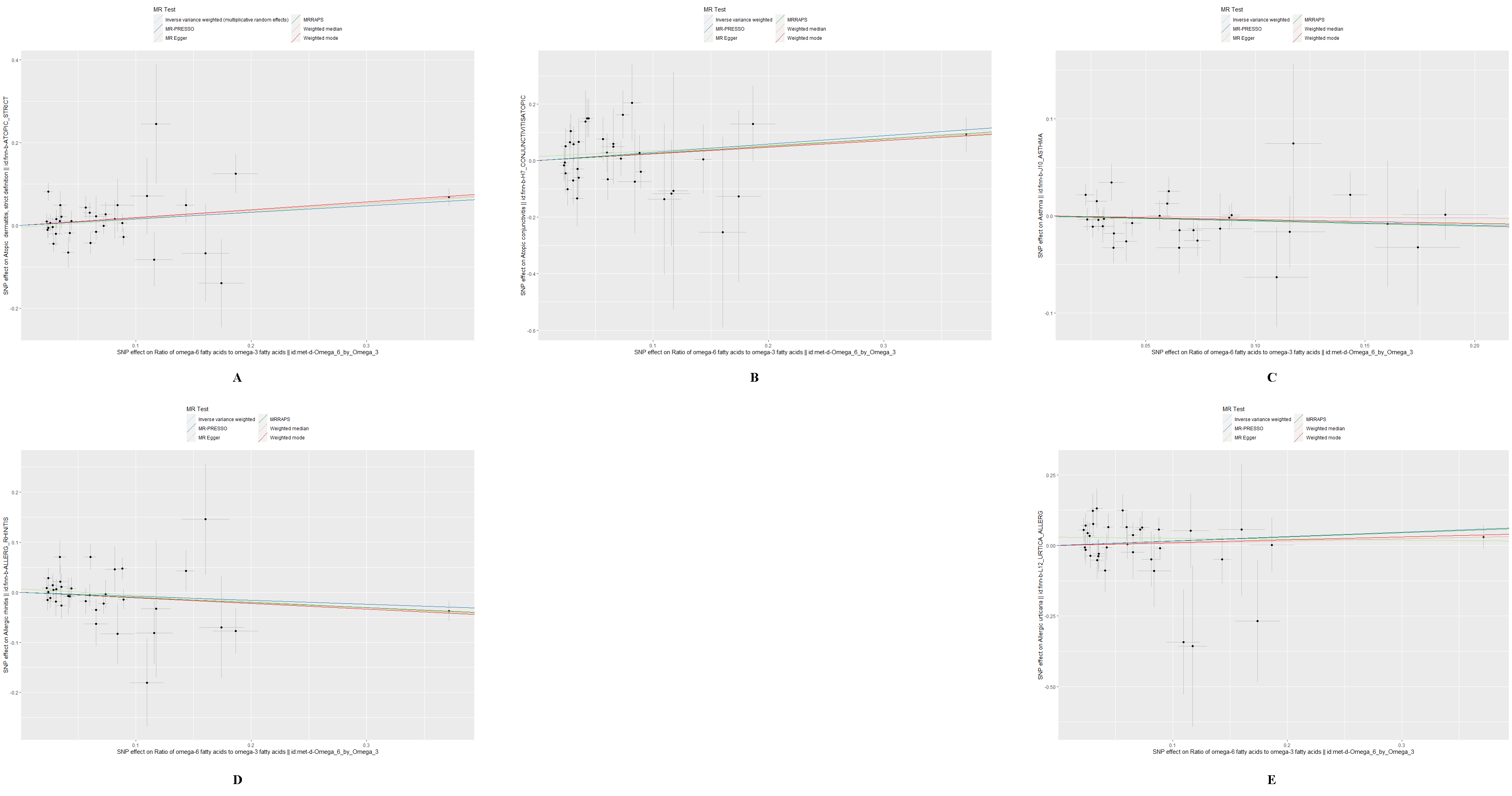


**Supplementary Figure 7.** Scatter plots of causal estimates of exposure (Ratio of omega-6 fatty acids to omega-3 fatty acids) on outcomes. The slope of each line corresponding to the estimated MR effect in different models, including the conventional IVW, MBE, WMM, MR-Egger, MR-RAPS and MR-PRESSO methods. The effect of A: AD; B: AC; C: Asthma; D: AR; E: AU. AD: atopic dermatitis; AC: Atopic conjunctivitis; AR: Allergic rhinitis; AU: Allergic urticaria.


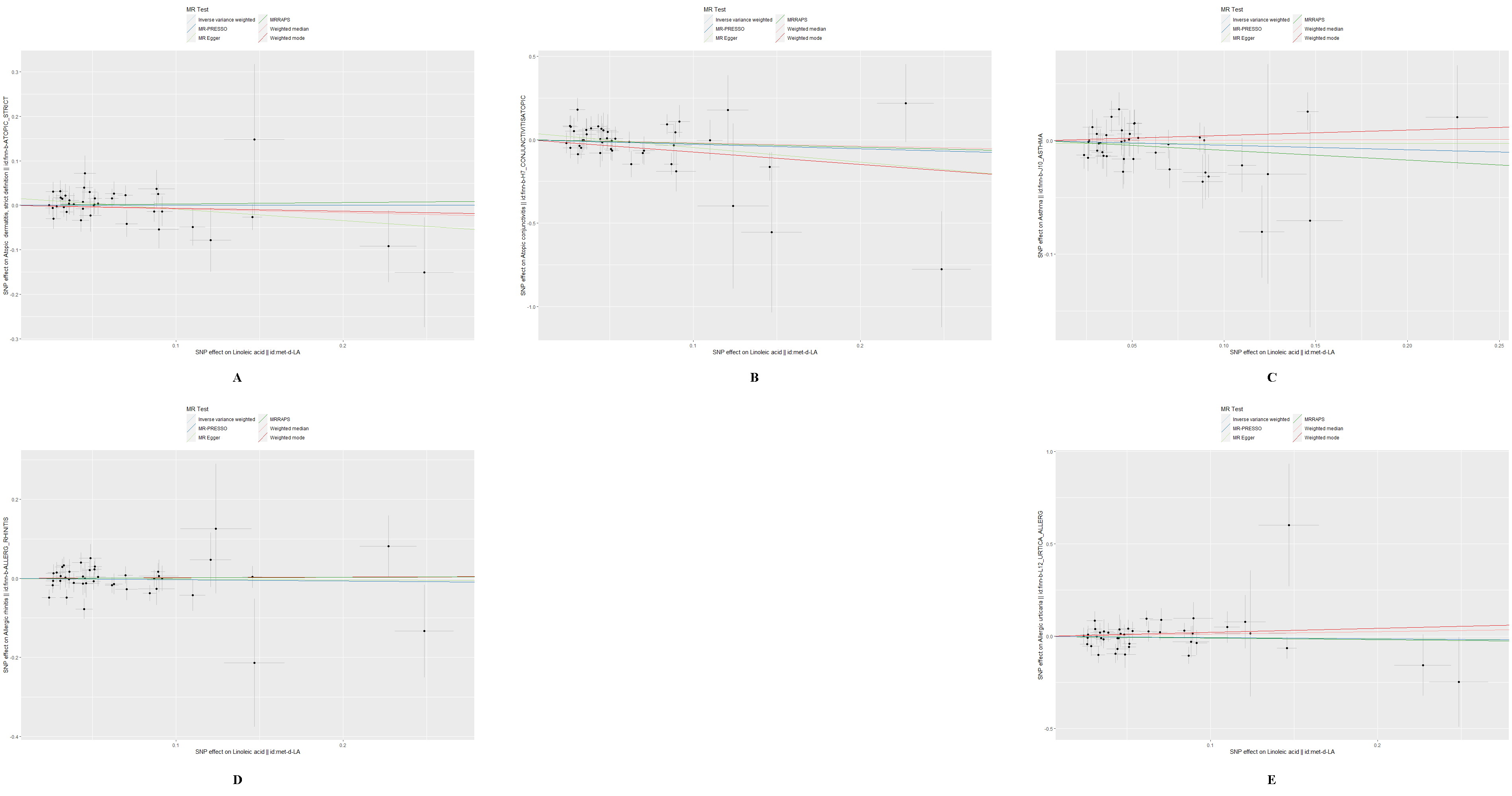


**Supplementary Figure 8.** Scatter plots of causal estimates of exposure (Linoleic acid) on outcomes. The slope of each line corresponding to the estimated MR effect in different models, including the conventional IVW, MBE, WMM, MR-Egger, MR-RAPS and MR-PRESSO methods. The effect of A: AD; B: AC; C: Asthma; D: AR; E: AU. AD: atopic dermatitis; AC: Atopic conjunctivitis; AR: Allergic rhinitis; AU: Allergic urticaria.


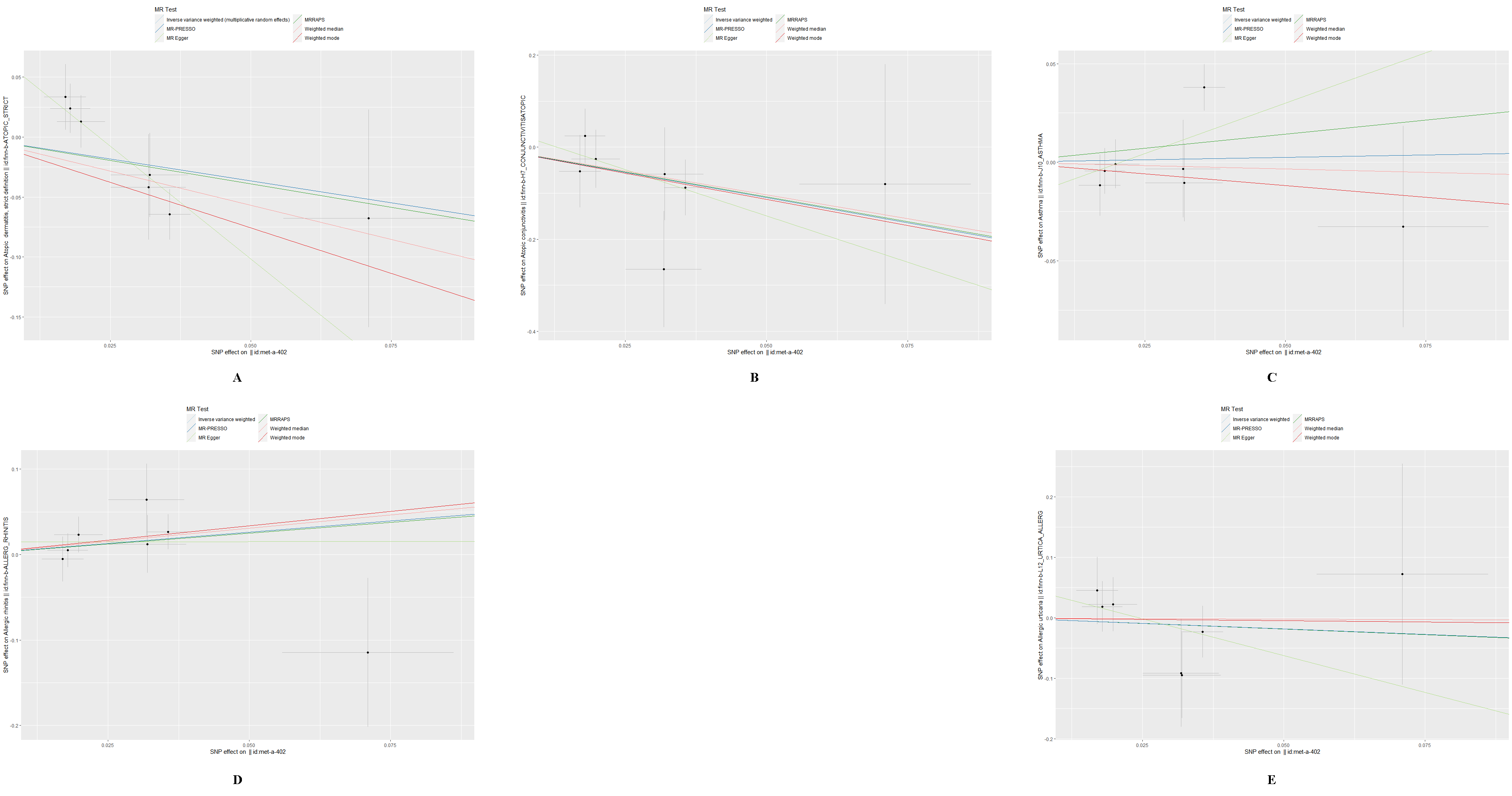


**Supplementary Figure 9.** Scatter plots of causal estimates of exposure (Eicosapentaenoic acid) on outcomes. The slope of each line corresponding to the estimated MR effect in different models, including the conventional IVW, MBE, WMM, MR-Egger, MR-RAPS and MR-PRESSO methods. The effect of A: AD; B: AC; C: Asthma; D: AR; E: AU. AD: atopic dermatitis; AC: Atopic conjunctivitis; AR: Allergic rhinitis; AU: Allergic urticaria.


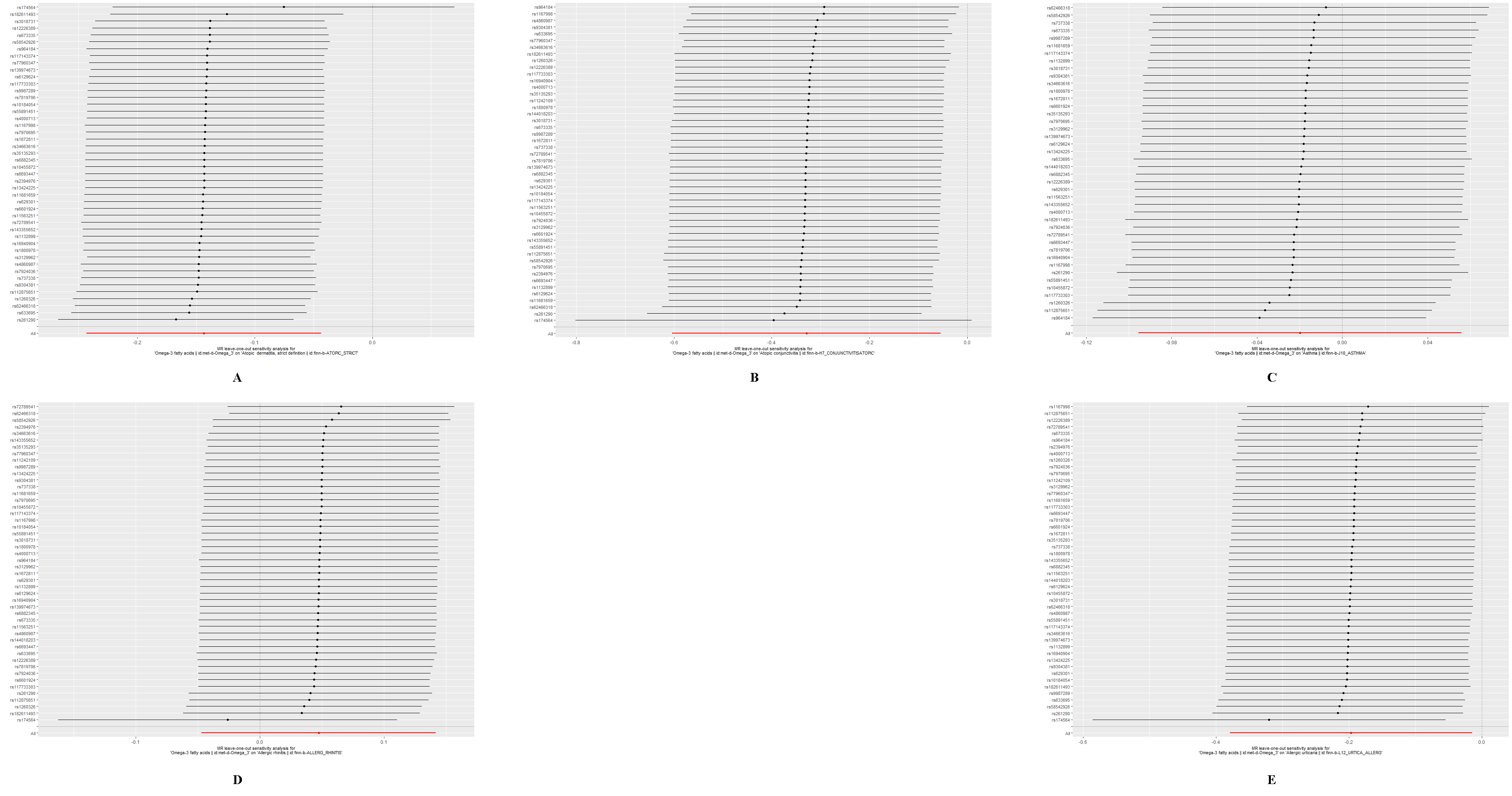


**Supplementary Figure 10.** Leave-one-out stability tests causal estimates of exposure (Omega-3 fatty acids) on outcomes. Calculate the MR results of the remaining IVs after removing the IVs one by one. The effect of A: AD; B: AC; C: Asthma; D: AR; E: AU. AD: atopic dermatitis; AC: Atopic conjunctivitis; AR: Allergic rhinitis; AU: Allergic urticaria.

.


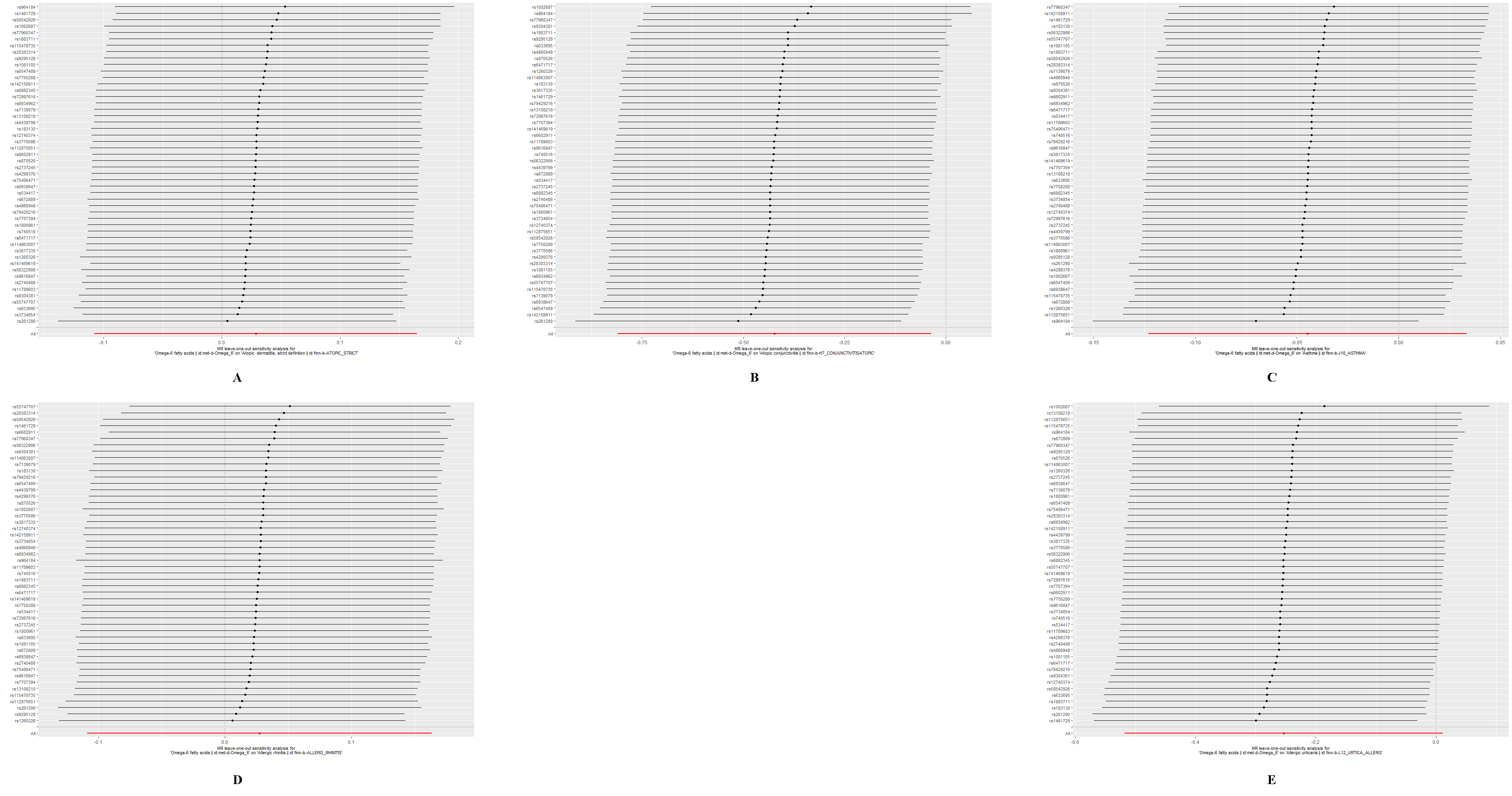


**Supplementary Figure 11.** Leave-one-out stability tests causal estimates of exposure (Omega-6 fatty acids) on outcomes. Calculate the MR results of the remaining IVs after removing the IVs one by one. The effect of A: AD; B: AC; C: Asthma; D: AR; E: AU. AD: atopic dermatitis; AC: Atopic conjunctivitis; AR: Allergic rhinitis; AU: Allergic urticaria.


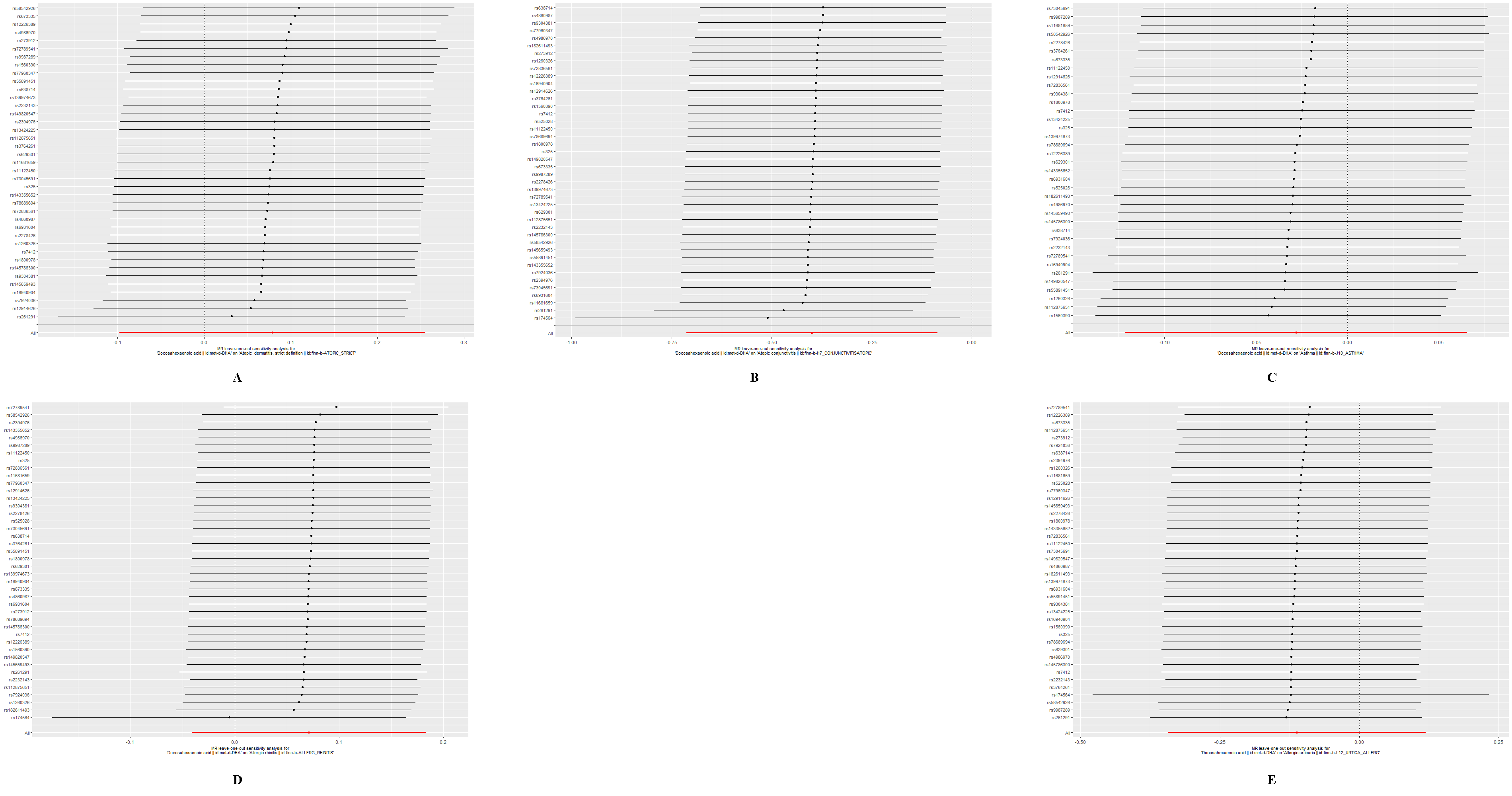


**Supplementary Figure 12.** Leave-one-out stability tests causal estimates of exposure (Docosahexaenoic acid) on outcomes. Calculate the MR results of the remaining IVs after removing the IVs one by one. The effect of A: AD; B: AC; C: Asthma; D: AR; E: AU. AD: atopic dermatitis; AC: Atopic conjunctivitis; AR: Allergic rhinitis; AU: Allergic urticaria.


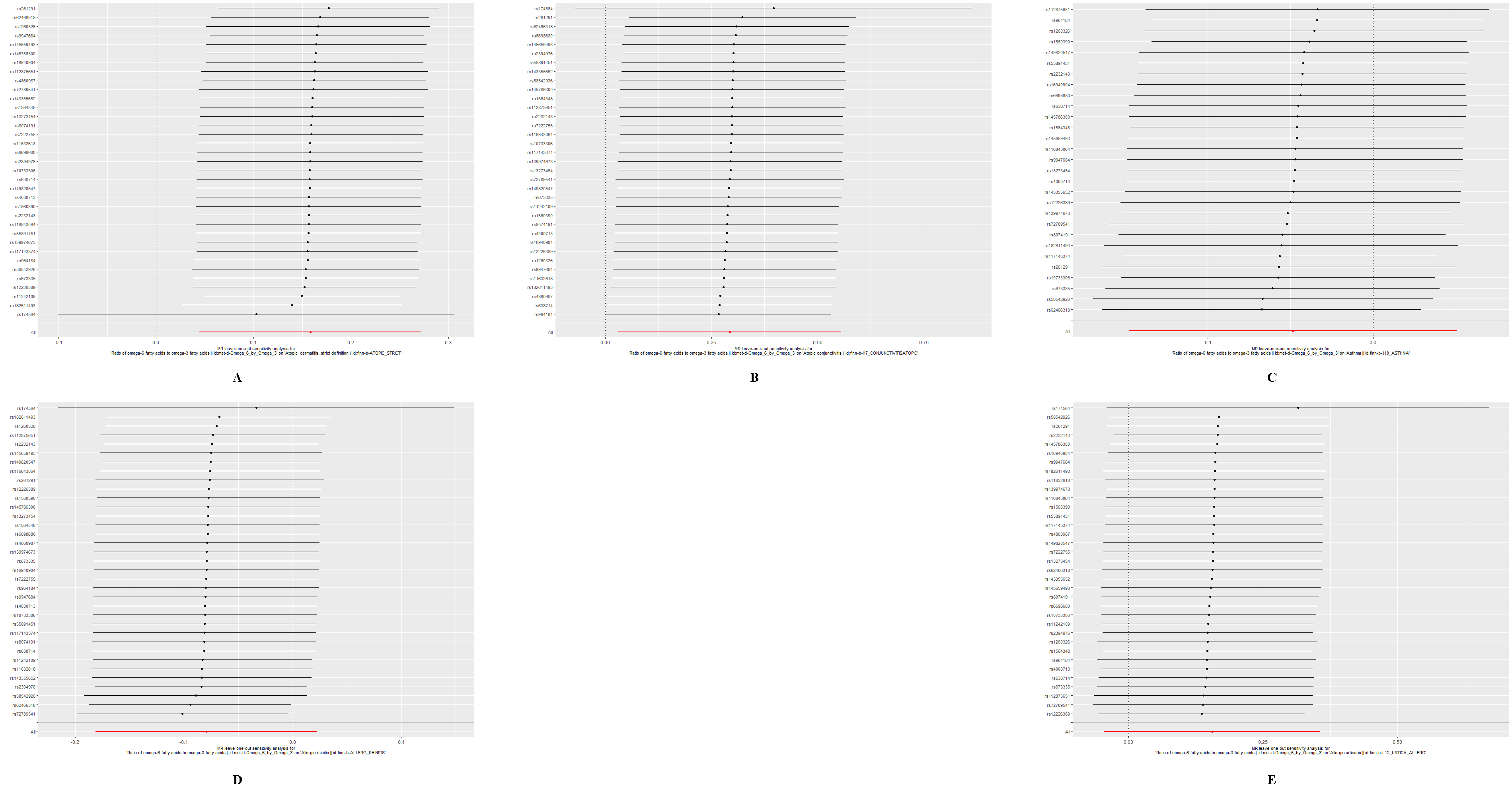


**Supplementary Figure 13.** Leave-one-out stability tests causal estimates of exposure (Ratio of omega-6 fatty acids to omega-3 fatty acids) on outcomes. Calculate the MR results of the remaining IVs after removing the IVs one by one. The effect of A: AD; B: AC; C: Asthma; D: AR; E: AU. AD: atopic dermatitis; AC: Atopic conjunctivitis; AR: Allergic rhinitis; AU: Allergic urticaria.


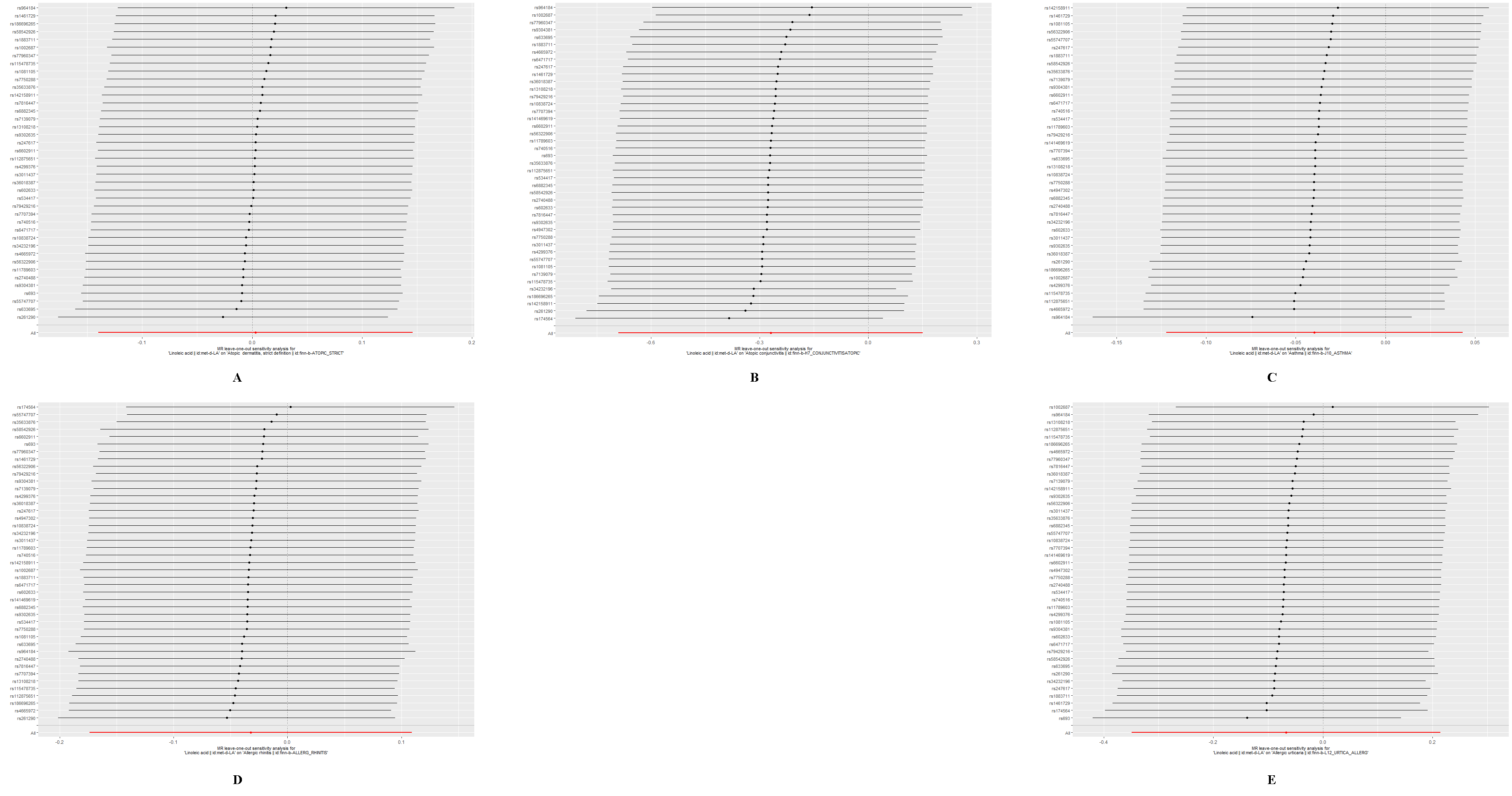
**Supplementary Figure 14.** Leave-one-out stability tests causal estimates of exposure (Linoleic acid) on outcomes. Calculate the MR results of the remaining IVs after removing the IVs one by one. The effect of A: AD; B: AC; C: Asthma; D: AR; E: AU. AD: atopic dermatitis; AC: Atopic conjunctivitis; AR: Allergic rhinitis; AU: Allergic urticaria.


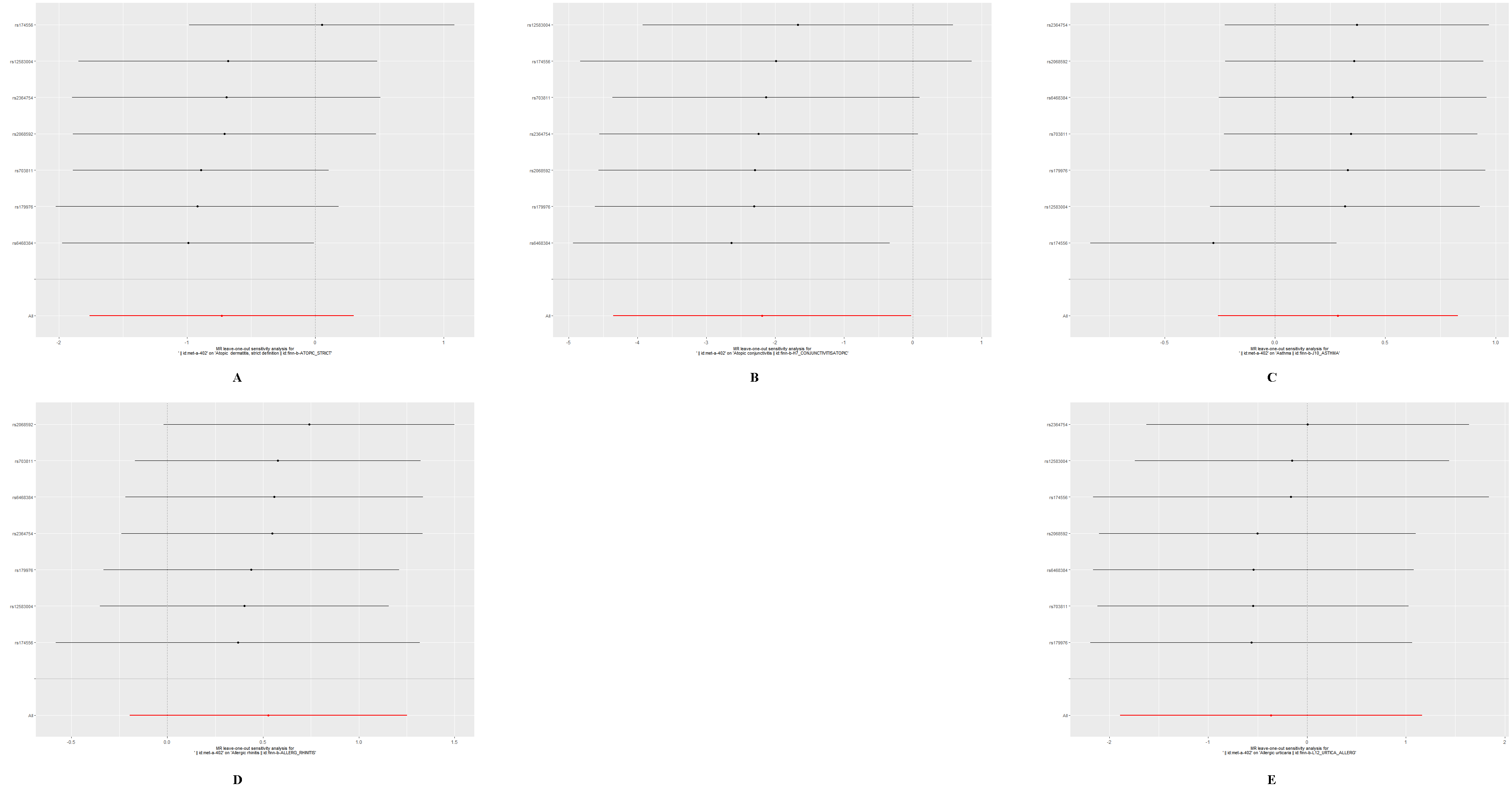


**Supplementary Figure 15.** Leave-one-out stability tests causal estimates of exposure (Eicosapentaenoic acid) on outcomes. Calculate the MR results of the remaining IVs after removing the IVs one by one. The effect of A: AD; B: AC; C: Asthma; D: AR; E: AU. AD: atopic dermatitis; AC: Atopic conjunctivitis; AR: Allergic rhinitis; AU: Allergic urticaria.
